# From Genome-Wide SNPs to Neuroimmune Crosstalk: Mapping the Genetic Landscape of IBD and Its Brain Overlap

**DOI:** 10.3390/biology14101433

**Published:** 2025-10-17

**Authors:** Utkarsh Tripathi, Yam Stern, Inbal Dagan, Ritu Nayak, Eva Romanovsky, Eran Zittan, Shani Stern

**Affiliations:** 1 Sagol Department of Neurobiology, Faculty of Natural Sciences, University of Haifa, Haifa 3498838, Israel; utripath@campus.haifa.ac.il (U.T.);; 2 The Rappaport Faculty of Medicine, Technion-Israel Institute of Technology, Haifa 3525433, Israel

**Keywords:** inflammatory bowel disease, Crohn’s disease, ulcerative colitis, genome-wide association studies, genotype-tissue expression, gut–brain axis, single-nucleotide polymorphisms, neuro-immune interactions, precision medicine

## Abstract

**Simple Summary:**

Inflammatory bowel disease (IBD), which includes Crohn’s disease and ulcerative colitis, is a long-term illness that causes inflammation in the gut. Scientists have found that IBD is not only a disease of the intestines but can also affect the brain, leading to problems such as depression, anxiety, and other neurological conditions. In this study, we examined genetic data from large international studies to identify genes that increase the risk of IBD. We then explored how these genes are expressed in both the gut and the brain. Our results showed that many of the same genes active in IBD are also active in specific brain regions and are involved in pathways that regulate immunity, stress response, and nerve function. This suggests that the genetic background of IBD may also influence brain health. By highlighting these shared pathways, our findings provide insights into why people with IBD are more likely to experience neurological or psychiatric problems. This knowledge could help guide new treatments, not only for IBD but also for related brain conditions and may eventually improve patient care by addressing the disease in a more holistic way.

**Abstract:**

Inflammatory bowel disease (IBD), comprising Crohn’s disease (CD) and ulcerative colitis (UC), arises from complex genetic and environmental interactions. Here, we integrate genome-wide association study (GWAS) meta-analyses with tissue-specific expression data from GTEx to map the polygenic architecture of IBD and its systemic implications. We identified 69 genome-wide significant single-nucleotide polymorphisms (SNPs) across 26 genes shared by CD and UC, revealing an almost equal partition of subtype-specific (50.7%) and shared (49.3%) risk variants. *IL23R* exhibited the highest allelic heterogeneity—three UC-specific, one CD-specific, and three shared SNPs—while *ATG16L1*′s four CD-specific variants underscored autophagy’s pivotal role in CD. Chromosomal mapping revealed distinct regulatory hotspots: UC-only loci on chromosomes 1 and 6, CD-only loci on chromosomes 10 and 16, and shared loci on chromosomes 7 and 19. Pathway enrichment emphasized IL-23/IL-17, Th17 differentiation, NF-κB, and JAK–STAT signaling as central to IBD pathogenesis. GTEx analyses showed uniformly high expression of IBD genes in gastrointestinal tissues, but pronounced heterogeneity across brain regions, including the cerebellum, frontal cortex, and hippocampus. This neuro-expression, together with enrichment of neurotrophin and neurodegeneration pathways and a nearly two-fold gene overlap with autism spectrum disorder, schizophrenia, and depression (FDR < 0.05), provides integrative evidence for gut–brain axis involvement in IBD. These findings consolidate prior work while extending perspectives on systemic disease implications. This study consolidates and systematizes dispersed genetic and transcriptomic findings into a unified reference framework. Our results highlight recurrent immune-regulatory and neuro-inflammatory pathways shared between gut and brain, offering a resource to guide future mechanistic, clinical, and translational investigations in IBD and related disorders.

## 1. Introduction

Inflammatory Bowel Disease (IBD) is a chronic, relapsing inflammatory condition of the gastrointestinal tract (GIT), primarily encompassing Crohn’s Disease (CD) and Ulcerative Colitis (UC). Despite extensive research, the precise etiology of IBD remains elusive, though it is widely accepted to be a multifactorial disorder influenced by genetic, environmental, and immunological factors [1,2]. The identification of genetic loci associated with IBD through genome-wide association studies (GWAS) has significantly advanced our understanding of the disease, with large-scale meta-analyses identifying over 200 susceptibility loci across diverse populations [3,4,5,6,7,8]. Recent high-resolution fine-mapping studies have further refined these associations to single-variant resolution [6,9]. However much remains to be uncovered about the functional implications of these genetic variations and their systemic effects beyond the gastrointestinal tract.

CD and UC, the two primary forms of IBD, present distinct clinical and pathological features, yet they share overlapping genetic and environmental risk factors. CD can affect any part of the GIT from mouth to anus, often leading to transmural inflammation, while UC is typically confined to the colon and rectum, with inflammation limited to the mucosal layer [10]. Despite these differences, both conditions are characterized by chronic inflammation, mucosal damage, and an increased risk of colorectal cancer [11].

Recent advances in high-throughput genomic technologies have enabled the comprehensive analysis of genetic and transcriptomic data, facilitating the identification of IBD-associated genes and their expression patterns across different tissues [12]. Complementing these technological advances, large-scale biobank resources such as the UK Biobank have enabled cross-trait genetic analyses that reveal shared genetic architectures between IBD and other complex diseases [13,14]. Modern analytical frameworks, including gene-set enrichment approaches and functional annotation methods [15,16], have transformed our ability to interpret GWAS findings in biological context. The integration of data from resources such as the Genotype-Tissue Expression (GTEx) project has further allowed for the exploration of gene expression in various tissues, including those outside the GIT, such as the brain [9,17]. This holistic approach provides valuable insights into the systemic nature of IBD and its potential impact on extra-intestinal tissues [4].

The brain–gut axis is the complex communication network linking the central nervous and gastrointestinal systems. The gut affects the brain through various mechanisms, notably the production of neurotransmitters and hormones. For example, the gut microbiota produces short-chain fatty acids that can influence the brain’s function and mood regulation [18]. Additionally, gut receptors can signal the brain about hunger and satiety, impacting emotional states and cognitive processes [19]. This relationship highlights the gut’s role as a vital component in mental health and cognition, indicating that gut health can directly influence emotional well-being through the microbiota-gut–brain axis.

Conversely, the brain can also significantly affect gut function through the autonomic nervous system. Stress and emotional disturbances can lead to intestinal motility and permeability changes, often leading to gastrointestinal symptoms. When the brain perceives stress, it can trigger the release of stress hormones such as cortisol, which may disrupt the gut’s normal function [20]. This communication pathway means that psychological states can manifest as physical symptoms in the gut, further underscoring the dynamic relationship between these two systems.

Mental stress is particularly relevant in the context of IBD, as it can exacerbate flares and increase symptoms [20]. Stressful events may lead to an imbalance in the gut microbiome and heightened inflammatory responses, which are critical factors in IBD. Many individuals with IBD report that high-stress moments correlate with worsening symptoms, such as abdominal pain and diarrhea. Managing stress through therapeutic interventions or lifestyle changes can, therefore, be an essential aspect of treating IBD, highlighting the importance of considering mental health in the overall management of gut-related disorders.

Moreover, the overlap between IBD and various neurological and psychiatric disorders, including schizophrenia, depression, autism spectrum disorder (ASD), and attention-deficit/hyperactivity disorder (ADHD), may suggests shared genetic and immunological pathways [14,21,22,23,24,25]. Studies show that depression and anxiety are more prevalent in individuals with IBD [26]. Some also suggest a potential association with Schizophrenia [27]. These comorbidities underscore the importance of understanding the genetic underpinnings of IBD not only within the context of gastrointestinal pathology but also concerning broader systemic and neurological health.

This review synthesizes current knowledge of the genetic landscape of IBD, CD, and UC by integrating GWAS data with GTEx expression profiles. We analyze expression patterns of IBD-associated genes across gastrointestinal and brain tissues and identify enriched biological pathways. In addition, we examine genetic overlap between IBD and several brain disorders, providing a systematic overview of shared architecture and possible common mechanisms. This integrative analysis advances understanding of IBD pathogenesis and its systemic implications, while highlighting opportunities for targeted therapeutic strategies and improved patient outcomes.

## 2. Materials and Methods

### 2.1. Data Collection and Preprocessing

#### 2.1.1. GWAS Data

Genome-wide association studies (GWAS) data for IBD, CD, and UC were obtained from publicly available databases. GWAS data for IBD and its subtypes were downloaded from the NHGRI-EBI GWAS Catalog on 2 November 2023, using trait identifier EFO_0007149 and child traits. The full dataset is available in Supplementary File S1 and includes study details (PUBMEDID, FIRST AUTHOR), sample sizes (INITIAL SAMPLE SIZE), and genetic associations. To avoid inflation from duplicate entries, we applied a unification procedure. If the same gene appeared multiple times within a single study, only the association with the most significant *p*-value was retained. This approach reduces bias from studies that report multiple independent associations for the same gene. The IBD dataset comprises 2700 associations from 207 studies, the CD dataset comprises 1092 associations from 71 studies, and the UC dataset comprises 801 associations from 66 studies. This provides a comprehensive overview of genetic variants linked to IBD and its subtypes. To ensure consistency in gene nomenclature across the GWAS data, the Human Gene Nomenclature Committee (HGNC) gene symbols were standardized using the HGNC database, which contains approved gene symbols, previous symbols, and aliases linked to Ensemble IDs. Following protocols described in previous studies [28,29], this standardization ensures accurate identification and comparison of genetic data across different datasets.

#### 2.1.2. GTEx Data

Expression data for IBD-associated genes were obtained from the Genotype-Tissue Expression (GTEx) project. The GTEx dataset provides comprehensive gene expression profiles across multiple human tissues. We specifically extracted expression data for gastrointestinal (GIT) tract tissues and various brain regions. The data included an Ensemble gene identifier (Gene), analyzed sample (Tissue), and normalized expression value; TPM (Transcripts Per Million) for each gene. TPM values are a normalization method for RNA-Seq data, accounting for the gene length and sequencing depth, allowing for accurate comparisons of gene expression levels across samples. We obtained TPM data from GTEx for all tissues, and extracted subsets for GIT regions (e.g., ileum, sigmoid colon, colon transverse) and 15 brain areas (e.g., cerebellum, frontal cortex, hippocampus). After mapping each IBD-associated gene to its Ensembl ID, we calculated the mean TPM per gene in each tissue group. To facilitate visualization, we ranked tissues by their average TPM and assigned colors (red = highest rank, violet = lowest rank) on a schematic diagram of the gut–brain axis. These heatmaps are descriptive and allow the visualization of the expression pattern in that specific tissue.

We had expression data for the following GIT areas: Colon-Sigmoid, Esophagus-Gastroesophageal Junction, Esophagus-Mucosa, Esophagus-Muscularis, Stomach, Colon-Transverse, and Small Intestine-Terminal Ileum. For brain regions, we had data from: the Amygdala, Anterior Cingulate Cortex (BA24), Caudate (basal ganglia), Cerebellar Hemisphere, Cerebellum, Cortex, Frontal Cortex (BA9), Hippocampus, Hypothalamus, Nucleus Accumbens (basal ganglia), Putamen (basal ganglia), Spinal Cord (cervical C1), and Substantia Nigra.

It is important to note that GTEx includes only non-diseased donor tissues; therefore, our analysis reflects baseline tissue and regional expression patterns, rather than disease-related dysregulation. TPM values across GTEx tissues can be influenced by sample size differences and tissue heterogeneity; cross-tissue comparisons should therefore be interpreted cautiously.

#### 2.1.3. Pathway Enrichment Analysis

For enrichment analyses, gene lists were generated based on the number of studies and associated SNPs from the GWAS Catalog datasets. Pathway enrichment analyses were conducted using standard enrichment tools (e.g., KEGG and Gene Ontology (GO) via ShinyGO 0.80 (March 2024). Pathways and gene ontology terms with false discovery rate (FDR)-adjusted *p*-values below 0.05 were considered significantly enriched.

#### 2.1.4. Brain and Signaling-Related Pathway Analysis

To explore the potential neurological implications of IBD-associated genes, we focused on brain-related pathways. We were specifically interested in KEGG pathways, which were directly related to brain or signaling, and these were plotted in addition to the general KEGG pathways. The identified pathways were visualized using bar plots to illustrate the fold enrichment and gene ratio for each pathway.

#### 2.1.5. Single Nucleotide Polymorphism (SNP) Analysis

We conducted a publication-based analysis to identify robustly associated genes and their single-nucleotide polymorphisms (SNPs) in ulcerative colitis (UC) and Crohn’s disease (CD). GWAS summary statistics were downloaded from the NHGRI–EBI GWAS Catalog (accessed 5 November 2023) for the traits “Ulcerative Colitis” (EFO_0000729) and “Crohn’s Disease” (EFO_0005624). From these files, we extracted SNP–gene pairs where chromosomal information (CHR_ID 1–24 and CHR_POS > 0) was valid, retaining 500 UC and 627 CD genome-wide significant SNP–gene associations (*p* < 5 × 10^−8^).

#### 2.1.6. SNP-to-Gene Mapping Strategy

To map SNPs to genes, we used the MAPPED_GENE and SNP_GENE_IDS columns from the GWAS Catalog. When multiple Ensemble gene IDs (ENSG) were associated with a single SNP entry, we unified them by gene symbol and counted the gene only once per publication to minimize noise from overlapping or redundant annotations. In cases where SNPs mapped to multiple genes (e.g., within gene clusters or shared loci), each gene was assigned independently but attributed once per publication. Chromosomal coordinates were verified and joined from the original summary statistics using a custom error-checked R script to ensure consistency in genomic localization.

We then curated a list of strongly associated genes based on their recurrence in multiple studies. Specifically, for each disease, gene–publication counts were derived from manually curated Excel sheets listing GWAS-reported genes along with the number of supporting publications. We defined strongly associated genes as those reported in ≥3 independent studies. Publication-based analysis identified 55 UC genes and 76 CD genes reported in ≥3 independent studies, with 26 genes shared between both conditions.

For these 26 common genes, we extracted all genome-wide significant SNPs (*p* < 5 × 10^−8^) and categorized each SNP–gene pair as “UC_only,” “CD_only,” or “Both” depending on its presence in the filtered datasets. This produced 69 SNPs in total: 10 exclusives to UC, 25 exclusive to CD, and 34 shared. All 69 SNPs were successfully mapped to chromosomal coordinates for genomic visualization.

Finally, we visualized the distribution of SNP types around the genome with circos plots (via RCircos v1.2.2) and mapped SNP association patterns across genes using an oncoprint matrix (via ComplexHeatmap v2.18.0).

#### 2.1.7. Word Cloud Generation

To visualize the prominence of genes associated with IBD, including Crohn’s disease (CD) and ulcerative colitis (UC), we generated gene-based word clouds derived from a custom MATLAB (vR2024a) and python (v3.9) pipeline. This pipeline integrates publication-derived *p*-values from the NHGRI–EBI GWAS Catalog (accessed 5 November 2023) and maps them to gene symbols using HGNC-compliant gene nomenclature. SNP-to-gene mappings were parsed using the MAPPED_GENE and SNP_GENE_IDS fields, followed by harmonization against HGNC base symbols, aliases, and previous names. If a gene appeared in multiple GWAS entries across different publications, all reported *p*-values were collected. For each gene, we computed the total number of GWAS entries with *p* < 0.01.

Genes were included in the word cloud if they were associated with any GWAS entry reporting a *p*-value < 0.01. Gene names were scaled in size according to this frequency count, visually highlighting the most frequently implicated genes.

#### 2.1.8. Genetic Overlap Analysis with Brain Disorders

We investigated the genetic overlap between IBD and several neurodevelopmental and psychiatric disorders, including schizophrenia (SCZ), autism spectrum disorder (ASD), attention-deficit/hyperactivity disorder (ADHD), and Depression. GWAS summary statistics for these brain disorders were retrieved from the NHGRI-EBI Genome-Wide Association Study (GWAS) Catalog in 15 April 2024. Specifically, data were downloaded for SCZ (EFO_0004609), ASD (EFO_0003757), ADHD (EFO_0003888), and Depression (EFO_0007453).

Our analysis included 87 GWAS studies comprising 1029 unique genes for IBD, 103 studies with 3193 genes for SCZ, 28 studies with 1062 genes for ASD, 43 studies with 1714 genes for ADHD, and 97 studies with 2473 genes for Depression (Supplementary Figure S1). Genes were extracted from the MAPPED_GENE column in the GWAS Catalog data, with multiple gene annotations per SNP parsed and deduplicated.

Statistical significance of genetic overlaps was assessed using Fisher’s exact test and hypergeometric enrichment analysis against a background of approximately 20,000 protein-coding genes in the human genome. We calculated odds ratios (OR) with 95% confidence intervals and enrichment ratios comparing observed to expected overlap under the null hypothesis of no association. Multiple testing correction was applied using the Benjamini–Hochberg false discovery rate (FDR) method. All statistical analyses were performed in R version 4.3.0, with significance set at FDR-adjusted *p* < 0.05.

#### 2.1.9. Overlap Among IBD Subtypes

The overlap between genes associated with IBD, CD, and UC was examined. Venn diagrams were used to illustrate the shared and unique genes among these subtypes. Additionally, an UpSet plot was generated to provide a detailed view of the intersections between IBD subtypes and brain disorders, allowing for the identification of genes common to multiple conditions.

### 2.2. Gene Expression Analysis

#### 2.2.1. Average Gene Expression Calculation

Expression data were analyzed using GTEx v8 RNA-seq data (*n* = 56,200 total genes, 413 IBD genes, 55,787 non-IBD genes). TPM values were used directly without any further transformation for descriptive analyses and visualization, as TPM values provide normalized expression levels suitable for cross-tissue comparison. For IBD-associated genes, the average expression levels across different GIT and brain tissues were calculated. This involved computing each tissue type’s mean TPM values for IBD genes. The resulting expression profiles were visualized using heat maps to highlight the distribution of IBD gene expression in the different tissues.

Biorender.com was utilized to assign color-coding based on the ranking of average expression level in the brain and GIT, with red signifying a high expression level and violet representing a low expression level. The illustration is not a precise representation of the anatomical structure of the brain and GIT but rather a schematic one. The colored areas were used to demonstrate the different brain and GIT regions.

#### 2.2.2. Visualization

All visualizations, including heatmaps, bar plots and UpSet plots, were generated using the R (v4.3.0) and Python 3.9 with following packages: ggplot2 (version 3.4.2), ComplexHeatmap (version 2.18.0), and UpSetR (version 1.4.0). Venn diagrams were plotted using online software MolBioTools (https://molbiotools.com/ accessed on 1 October 2025). The visualizations were designed to provide clear and comprehensive representations of the data, facilitating the interpretation of gene expression patterns and pathway enrichment results.

## 3. Results

### 3.1. Genetic Architecture of IBD Reveals Key Susceptibility Loci

**Figure 1:**
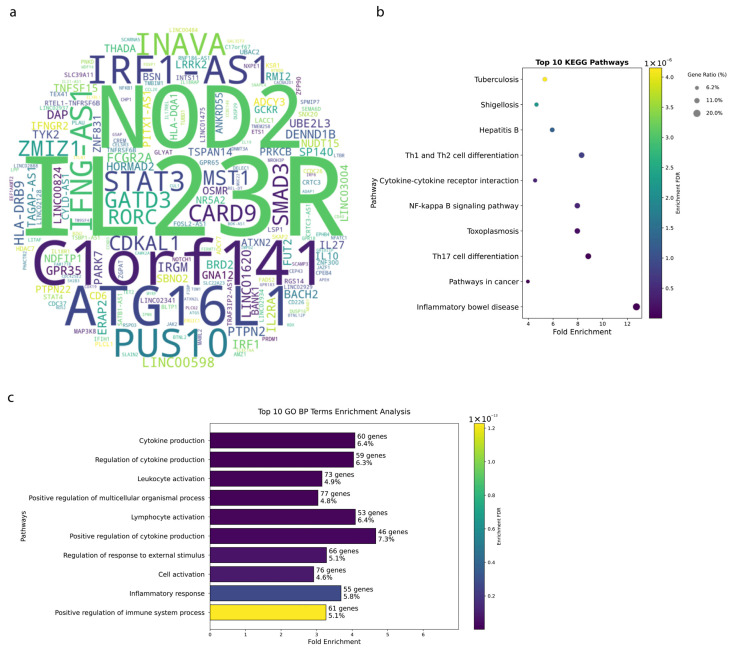
Genetic Associations in Inflammatory Bowel Disease (IBD) (**a**) Word-cloud of 1029 IBD-associated genes (GWAS *p* < 0.01), with name size proportional to publication frequency. (**b**) Top 10 KEGG pathways enriched among these genes (FDR < 1 × 10^−6^), highlighting core immune and barrier functions in IBD. (**c**) Top 10 GO Biological Processes (FDR < 1 × 10^−6^), illustrating dominant roles in cytokine signaling and leukocyte activation.

The genetic architecture of IBD encompasses a wide array of genes that contribute to the complex etiology of the disease. Through comprehensive analysis of GWAS data from 207 IBD-related studies, we identified key genetic determinants with varying frequencies of association. As shown in Figure 1a, the word cloud reveals the most frequently implicated genes, with gene name size proportional to publication frequency (see Methods).

*IL23R* emerged as the most consistently reported gene (38 publications), reflecting its critical role in Th17 cell differentiation and IL-23 signaling. *NOD2*, appearing in 22 publications, represents a well-established pattern recognition receptor crucial for bacterial sensing. Following these, *PUS10*, *IRF1*, *ATG16L1*, and *C1orf141* each appeared in 15 publications, highlighting their significant contributions to IBD susceptibility. To explore the functional implications of these genetic associations, we further conducted pathway enrichment analyses. The top 10 KEGG pathways (Figure 1b) showed striking enrichment, with the Inflammatory Bowel Disease pathway demonstrating the strongest signal (13 genes, FDR = 2.8 × 10^−9^, nFold = 12.74). Notably, the Th17 Cell Differentiation pathway (15 genes, FDR = 1.3 × 10^−8^, nFold = 8.84) and NF-kappa B Signaling Pathway (13 genes, FDR = 4.0 × 10^−7^, nFold = 7.96) showed robust enrichment, highlighting their central roles in IBD pathophysiology. The top 10 KEGG pathways showed strong enrichment, including IBD (13 genes, FDR = 2.8 × 10^−9^, nFold = 12.74), Th17 Cell Differentiation (15 genes, FDR = 1.3 × 10^−8^, nFold = 8.84), and NF-κB Signaling (13 genes, FDR = 4.0 × 10^−7^, nFold = 7.96). Cytokine–cytokine receptor interactions, as well as Th1/Th2 differentiation pathways, were also significantly enriched.

The Gene Ontology analysis (Figure 1c) revealed that biological processes related to immune cell function dominate the IBD genetic landscape. The most enriched terms include Leukocyte Activation (73 genes, FDR = 1.3 × 10^−15^, nFold = 3.15), Regulation of Cytokine Production (59 genes, FDR = 9.7 × 10^−17^, nFold = 4.04), Positive Regulation of Multicellular Organismal Process (77 genes, FDR = 1.3 × 10^−15^, nFold = 3.04), Lymphocyte Activation (53 genes, FDR = 2.3 × 10^−15^nFold = 4.09), and Positive Regulation of Immune System Process (61 genes, FDR = 1.2 × 10^−13^, nFold = 3.26). These biological processes are essential for the initiation and maintenance of immune responses.

### 3.2. Crohn’s Disease Exhibits Distinct Genetic Signatures

**Figure 2:**
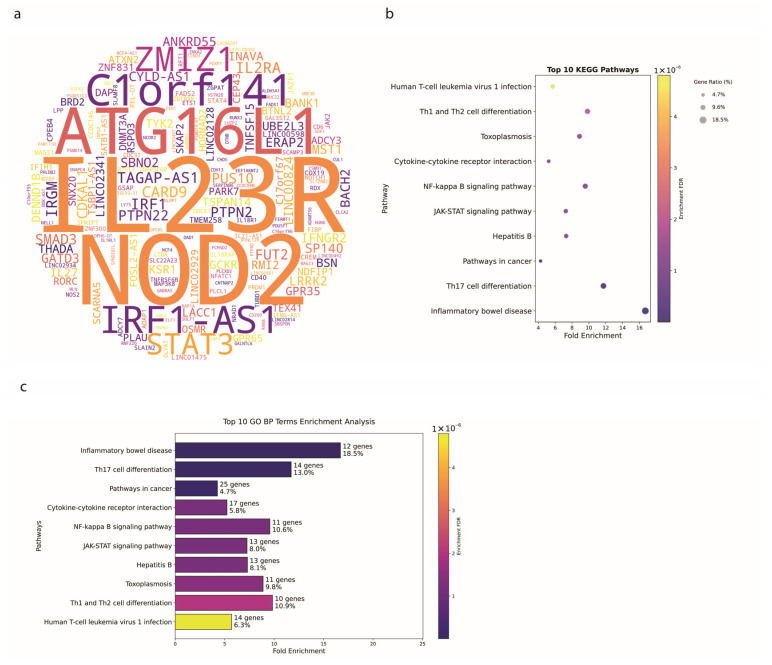
Genetic Associations in CD (**a**) A word cloud of genes associated with CD. The size of each gene’s name reflects its frequency of mention in the literature. Prominent genes include *NOD2*, *IL23R*, *C1orf141*, *ATG16L1*, and *IRF1-AS1*. (**b**) The top 10 enriched KEGG pathways in CD-associated genes. Significant pathways include the Inflammatory Bowel Disease Pathway, Th17 Cell Differentiation, and JAK-STAT Signaling Pathway. (**c**) The top 10 enriched GO Biological Process terms in CD GWAS data. Enriched terms include inflammatory response, Cytokine Production, and Positive Regulation of signal transduction.

To delve deeper into the genetic underpinnings specific to CD, we examined a separate subset of the GWAS data that is specific to CD. Figure 2a shows a word cloud of genes associated with CD. Prominent genes include *IL23R*, *NOD2*, *ATG16L1*, *C1orf141*, and *IRF1-AS1*, consistent with those identified in the broader IBD analysis, underscoring their critical roles in the disease. Notably, *IL23R* appears in 21 publications, making it the most significant gene, followed by *NOD2* (18 publications), *ATG16L1* (13 publications), *C1orf141* (12 publications), and *IRF1-AS1* (10 publications).

The pathway enrichment analyses for CD, as depicted in Figure 2b, reveal significant insights into the functional implications of these genetic associations. The most significantly enriched pathways include the Inflammatory Bowel Disease (12 genes, FDR = 1.5 × 10^−9^, nFold = 16.7), Th17 Cell Differentiation (14 genes, FDR = 1.9 × 10^−9^, nFold = 11.73), NF-Kappa B Signaling Pathway (11 genes, FDR = 1.2 × 10^−6^, nFold = 9.57), Cytokine-Cytokine Receptor Interaction (17 genes, FDR = 1.2 × 10^−6^, nFold = 5.23), and The JAK-STAT Signaling Pathway (13 genes, FDR = 1.2 × 10^−6^, nFold = 7.26).

Gene Ontology analysis for CD (Figure 2c) emphasized the centrality of cytokine networks, with both “Cytokine Production” and “Regulation of Cytokine Production” showing identical enrichment statistics (49 genes each, FDR = 1.5 × 10^−16^, nFold = 4.73 and 4.77, respectively), suggesting tight regulatory control of inflammatory mediators in CD pathogenesis.

The enrichment of BP terms like Cytokine Production and Regulation of Cytokine Production suggests a possible central role of cytokine networks in CD pathophysiology.

These findings provide a detailed understanding of the genetic and molecular landscape specific to CD, emphasizing the significant overlap in genetic and pathway associations with the broader IBD category while highlighting specific processes and signaling pathways that contribute uniquely to CD pathology.

### 3.3. Ulcerative Colitis Shows Tissue-Specific Genetic Patterns

**Figure 3:**
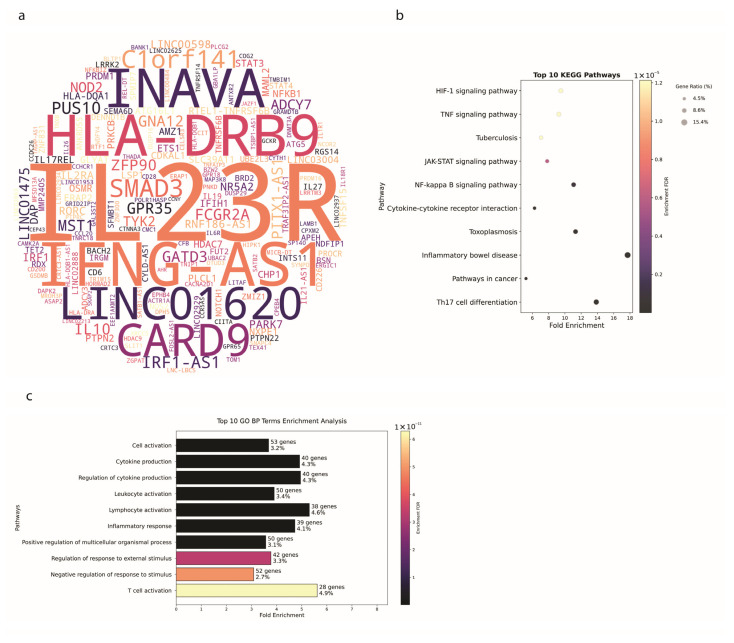
Genetic Associations in Ulcerative Colitis (**a**) A word cloud of genes associated with UC. The size of each gene’s name reflects its frequency of mention in the literature. Prominent genes include *IL23R*, *HLA-DRB9*, *CARD9*, *IFNG-AS1*, and *INAVA*. (**b**) The top 10 enriched KEGG pathways in UC-associated genes. Significant pathways include the HIF-1 Signaling Pathway, TNF Signaling Pathway, and JAK-STAT Signaling Pathway. (**c**) The top 10 enriched GO Biological Process terms in UC GWAS data. Enriched terms include Cell Activation, Cytokine Production, and Regulation of Cytokine Production.

Continuing from the genetic insights gained from the broader IBD and CD analyses, we now focus on UC. Figure 3a displays a word cloud of genes associated with UC, with the size of each gene’s name reflecting its frequency in the GWAS data. Prominent genes include *IL23R* (17 publications), *IFNG-AS1* (9 publications), *HLA-DRB9* (8 publications), *INAVA* (7 publications), *CARD9* (7 publications), and *LINC01620* (7 publications). Notably, *IL23R* is observed across all 3 conditions: IBD (38 publications), CD (21 publications), and UC. Additionally, *NOD2* and *ATG16L1*, which are highly prominent in IBD (22 and 15 publications, respectively) and CD (18 and 13 publications, respectively), were observed in only 4 and 3 publications in UC, respectively, indicating a lesser but still notable involvement in UC pathogenesis.

The pathway enrichment analyses for UC, illustrated in Figure 3b, reveal significant insights into the functional implications of these genetic associations. HIF-1 Signaling pathway (9 genes, FDR = 1.2 × 10^−5^, nFold = 9.5), TNF Signaling Pathway (9 genes, FDR = 1.2 × 10^−5^, nFold = 9.25), JAK-STAT Signaling Pathway (11 genes, FDR = 5.9 × 10^−6^, nFold = 7.8), NF-Kappa B Signaling Pathway (10 genes, FDR = 9.3 × 10^−7^, nFold = 11.07), and Cytokine-Cytokine Receptor Interaction (16 genes, FDR = 3.0 × 10^−7^, nFold = 16). These pathways highlight both common and unique aspects of UC pathogenesis compared to CD and the broader IBD category.

The NF-Kappa B Signaling Pathway and Cytokine-Cytokine Receptor Interaction pathways are also shared between UC and CD. These pathways are integral to the broader IBD pathology, underscoring the possible shared mechanisms underlying these diseases.

The top 10 Gene Ontology (GO) Biological Process (Figure 3c) terms enriched in the UC GWAS data include Cell Activation (53 genes, FDR = 3.6 × 10^−14^, nFold = 3.68), Cytokine Production (40 genes, FDR = 3.6 × 10^−14^, nFold = 4.91), Regulation of Cytokine Production (40 genes, FDR = 3.6 × 10^−14^, nFold = 4.95), Leukocyte Activation (50 genes, FDR = 3.6 × 10^−14^, nFold = 3.90), and Inflammatory Response (39 genes, FDR = 2.9 × 10^−13^, nFold = 4.73).

The enrichment of Lymphocyte Activation (38 genes, FDR = 3.6 × 10^−14^, nFold = 5.30) is shared across UC, CD, and the broader IBD category, underscoring the role of adaptive immune responses in these conditions. The Positive Regulation of Multicellular Organismal Process (50 genes, FDR = 7.9 × 10^−13^, nFold = 3.57) and Regulation of Response to External Stimulus (42 genes, FDR = 3.3 × 10^−11^, nFold = 3.77) terms further highlight the complex interactions between immune cells and their environment in UC.

The Negative Regulation of Response to Stimulus (32 genes) and T Cell Activation (28 genes) terms reflect the intricate balance of immune activation and regulation required to maintain homeostasis and prevent excessive inflammation in UC. These terms are also enriched in CD, indicating potential shared regulatory mechanisms in these IBD subtypes.

### 3.4. IBD-Associated Genes Show Differential Expression Patterns Along the Gut–Brain Axis

To explore the systemic implications of IBD genetics, we analyzed expression patterns of 413 IBD-associated genes across 54 tissue types from the GTEx database, focusing on 13 brain regions and 7 gastrointestinal regions.

Overall Expression Patterns: Our analysis revealed that IBD-associated genes exhibited significantly higher mean expression levels in GIT regions (22.54 TPM) compared to brain regions (12.76 TPM; Mann–Whitney U test, *p* = 2.04 × 10^−4^) (Supplementary Figure S3). This finding aligns with the expected primary role of these genes in gastrointestinal pathophysiology. When comparing IBD genes to non-IBD genes within each tissue group, we observed highly significant differences across all categories: brain tissues (*p* = 9.39 × 10^−82^), GIT tissues (*p* = 2.65 × 10^−97^), and other tissues (*p* = 1.17 × 10^−90^), indicating that IBD-associated genes have distinct expression profiles compared to background genes.

Interestingly, while IBD genes showed higher overall expression in GIT regions, we observed striking differences in regional expression heterogeneity. IBD genes demonstrated significant expression variation across brain regions (ANOVA *p* = 6.37 × 10^−9^) but not across GIT regions (ANOVA *p* = 0.339) (Supplementary Figure S3). This unexpected finding may suggest that IBD-associated genes may have more specialized, region-specific roles in neural tissues compared to their more uniform expression patterns in the GIT. While many genes in the genome are indeed expressed at some level in brain tissue, our analysis focused on relative regional differences in the expression of IBD-associated genes. We observed significant enrichment in specific areas, such as the cerebellum and frontal cortex, compared to other regions, suggesting potential region-specific susceptibility to gut–brain axis influences.

**Figure 4:**
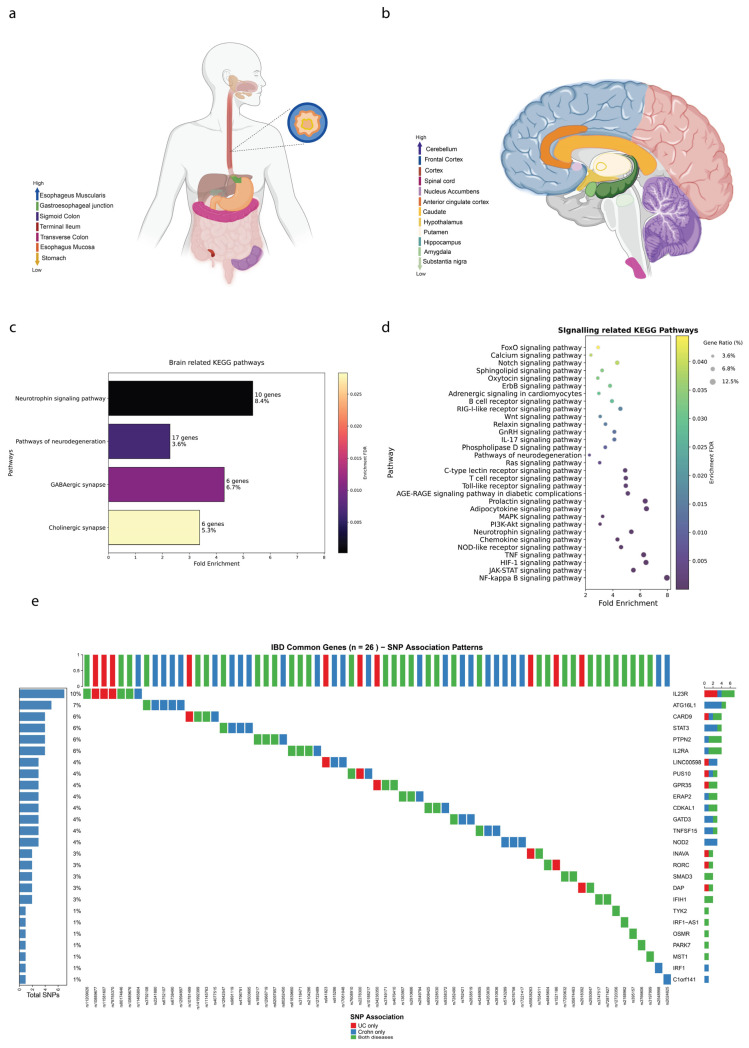
Expression Patterns and Genetic Architecture of IBD-Associated Genes Across Tissues (**a**) GIT Expression Heatmap. Average expression levels (TPM) of IBD-associated genes across seven regions of the GIT obtained from GTEx data. Tissues include gastroesophageal junction, sigmoid colon, terminal ileum, transverse colon, esophagus muscularis, esophagus mucosa, and stomach. Expression levels are color-coded from low (blue) to high (red). Gray indicates tissues for which expression data were not available. (**b**) Brain Tissue Expression Heatmap. Expression levels (TPM) of IBD-associated genes across twelve brain regions from GTEx data, including cerebellum, frontal cortex (BA9), cortex, spinal cord (cervical C1), nucleus accumbens (basal ganglia), anterior cingulate cortex (BA24), caudate (basal ganglia), hypothalamus, putamen (basal ganglia), hippocampus, amygdala, and substantia nigra. Expression levels are color-coded from low (blue) to high (red). Gray indicates brain regions for which expression data were not available. (**c**) Brain-Related KEGG Pathway Enrichment. Significantly enriched brain-related KEGG pathways among IBD-associated genes. Key pathways include neurotrophin signaling pathways and pathways of neurodegeneration, highlighting the neurological connections of IBD genetics. (**d**) Signaling-Related KEGG Pathway Enrichment. Significantly enriched signaling-related KEGG pathways among IBD-associated genes. Notable pathways include FoxO signaling pathway, calcium signaling pathway, and Notch signaling pathway, demonstrating the diverse cellular processes involved in IBD pathogenesis. (**e**) SNP Association Patterns in IBD Common Genes. OncoPrint visualization displaying the distribution of single nucleotide polymorphisms (SNPs) across 26 strongly IBD-associated genes identified through rigorous publication-based filtering. Each row represents a gene, and each column represents a SNP. Color coding indicates disease-specific association patterns: UC-only (orange), Crohn’s disease-only (blue), and shared between both conditions (green). The percentage values on the right indicate the proportion of total SNPs associated with each gene, with *IL23R* showing the highest genetic burden (10% of total SNPs) followed by *ATG16L1* (7% of total SNPs).

Figure 4a presents the mean TPM values for IBD-associated genes across gastrointestinal regions, ranked from highest to lowest expression. The esophagus muscularis (29.07 TPM), gastroesophageal junction (28.79 TPM), and sigmoid colon (25.32 TPM) exhibited the highest expression levels, underscoring their potential significance in IBD pathology. The relatively uniform expression across GIT regions (*p* = 0.339) may suggest these genes may have broadly conserved functions throughout the GIT.

Figure 4b ranks gene expression across brain regions, revealing more pronounced regional variation. The cerebellum (18.66 TPM), frontal cortex BA9 (15.54 TPM) and cortex (14.29 TPM) showed the highest expression levels, with significant differences between regions (*p* = 6.37 × 10^−9^). This heterogeneous expression pattern may suggest that IBD-associated genes may have specialized neurological functions that vary by brain region.

Several genes emerged as highly expressed in both tissue types, though with distinct expression patterns (Supplementary Figure S3). Among the top brain-expressed genes, *CAMK2A* showed the highest expression at 214.39 TPM, representing calcium/calmodulin-dependent protein kinase II alpha, which is crucial for synaptic plasticity and learning. Its high brain expression may suggest potential roles in IBD-associated cognitive symptoms and stress responses. *PARK7*, also known as *DJ-1*, demonstrated significant expression at 189.28 TPM and is involved in neuroprotection and oxidative stress response. Its dual expression in brain and GIT (158.75 TPM) may link oxidative stress mechanisms between the two systems. *DAD1*, or Defender against apoptotic death 1, showed expression levels of 122.18 TPM in brain tissue and is involved in protein trafficking and cell survival, with similarly high GIT expression at 181.20 TPM.

Among the top GIT-expressed genes, *ACTA2* demonstrated exceptionally high expression at 1446.43 TPM, representing alpha-smooth muscle actin, which is essential for smooth muscle contraction and wound healing. Its remarkably high GIT expression contrasts with minimal brain expression (10.2 TPM), reflecting its specialized role in gastrointestinal motility and fibrosis. *HLA-DRA*, encoding a major histocompatibility complex class II protein critical for immune recognition, showed high GIT expression at 283.76 TPM versus moderate brain expression at 75.2 TPM, underscoring the immune-mediated nature of IBD pathogenesis.

To further understand the functional implications of the transcriptomic data, we conducted pathway enrichment analyses. Figure 4c presents brain-related KEGG pathways enriched with IBD genes. Significant pathways include the Neurotrophin Signaling Pathway (10 genes, FDR = 1.9 × 10^−4^, nFold = 5.35, and the Pathways of Neurodegeneration (17 genes, FDR = 6.0 × 10^−3^, nFold = 2.28). Additionally, the GABAergic Synapse (6 genes, FDR = 1.1 × 10^−2^, nFold = 4.29) and Cholinergic Synapse (6 genes, FDR = 2.9 × 10^−2^, nFold = 3.38) pathways are enriched, reflecting the involvement of IBD genes in neurotransmitter signaling and synaptic function.

To complement the pathways highlighted in Figure 1b, additional signaling pathways were found to be enriched with IBD-associated genes and presented in Figure 4d, emphasizing the breadth of disturbed signaling networks in IBD. There were 41 enriched KEGG pathways that were related to signaling. The most enriched pathways include the NF-kappa B signaling pathway (nGenes = 13, FDR = 4.0 × 10^−7^, nFold = 7.96), JAK-STAT signaling pathway (nGenes = 14, FDR = 6.0 × 10^−6^, nFold = 5.50), HIF-1 signaling pathway (nGenes = 11, FDR = 1.9 × 10^−5^, nFold = 6.43), TNF signaling pathway (nGenes = 11, FDR = 2.3 × 10^−5^, nFold = 6.25), and NOD-like receptor signaling pathway (nGenes = 13, FDR = 7.3 × 10^−5^, nFold = 4.60).

Several enriched pathways were related to brain function and neurological processes. These included the Neurotrophin signaling pathway (10 genes, FDR = 1.9 × 10^−4^, Fold Enrichment = 5.35), the Oxytocin signaling pathway (7 genes, FDR = 3.3 × 10^−2^, Fold Enrichment = 2.89), the Calcium signaling pathway (9 genes, FDR = 3.9 × 10^−2^, Fold Enrichment = 2.39), the Sphingolipid signaling pathway (6 genes, FDR = 3.3 × 10^−2^, Fold Enrichment = 3.21), the Notch signaling pathway (4 genes, FDR = 3.9 × 10^−2^, Fold Enrichment = 4.32), and the Wnt signaling pathway (8 genes, FDR = 1.7 × 10^−2^, Fold Enrichment = 3.07).

### 3.5. Genetic Variant Architecture Reveals Disease-Specific and Shared Susceptibility Patterns

To comprehensively characterize the genetic architecture underlying IBD pathogenesis, we conducted a systematic analysis of single-nucleotide polymorphisms (SNPs) associated with the 26 shared IBD-associated genes. Figure 4e presents an OncoPrint visualization revealing the complex genetic landscape of IBD susceptibility variants. Our analysis identified 69 genome-wide significant SNPs distributed across the 26 IBD-associated genes, with striking heterogeneity in disease-specific associations. The genetic variants demonstrated three distinct association patterns: 10 SNPs (14.5%) exhibited exclusive association with UC, 25 SNPs (36.2%) were specifically associated with CD, and 34 SNPs (49.3%) showed shared associations with both IBD subtypes, indicating substantial genetic overlap between these clinically distinct conditions.

Gene-specific genetic burden analysis revealed considerable variation in SNP density across IBD-associated loci. *IL23R* emerged as the most genetically complex locus, harboring 7 associated SNPs (10.1% of total variants), followed by *ATG16L1* with 5 SNPs (7.2% of total). This genetic heterogeneity within individual loci underscores the multifactorial nature of IBD susceptibility. *IL23R* exemplifies the complex genetic architecture of IBD, containing three UC-specific variants (rs11209026, rs10889677, rs11581607), one CD-specific variant (rs79755370), and three shared variants (rs80174646, rs10889676, rs11465804), demonstrating how a single gene can contribute to both IBD subtypes through different molecular mechanisms. *ATG16L1* represents another paradigm of IBD genetic complexity, with four CD-specific variants and one shared variant.

The predominance of shared variants (49.3%) may suggest common pathogenic mechanisms between UC and CD, while the substantial proportion of disease-specific variants (50.7% combined) supports the concept of IBD as a spectrum of related but distinct inflammatory conditions. This genetic architecture provides molecular evidence for the clinical observation that UC and CD, while sharing inflammatory bowel pathology, have distinct pathophysiological mechanisms and therapeutic responses. Mapping these SNPs onto the genome through circos plots revealed distinct chromosomal hotspots: UC-specific variants predominantly on chromosomes 1 and 6, CD-specific on chromosomes 10 and 16, and shared SNPs concentrated on chromosomes 7 and 19 (Supplementary Figure S2). These genomic insights underscore the shared genetic architecture and distinct disease-specific variant patterns within IBD.

### 3.6. IBD Shows Significant Genetic Overlap with Neuropsychiatric Disorders

Expanding on our previous analyses, we examined the overlap between IBD-associated genes and genes implicated in various brain disorders. There were many previous reports of comorbidities between IBD and neurological conditions [24,30]. This analysis provides insights into the shared genetic architecture between these conditions.

**Figure 5:**
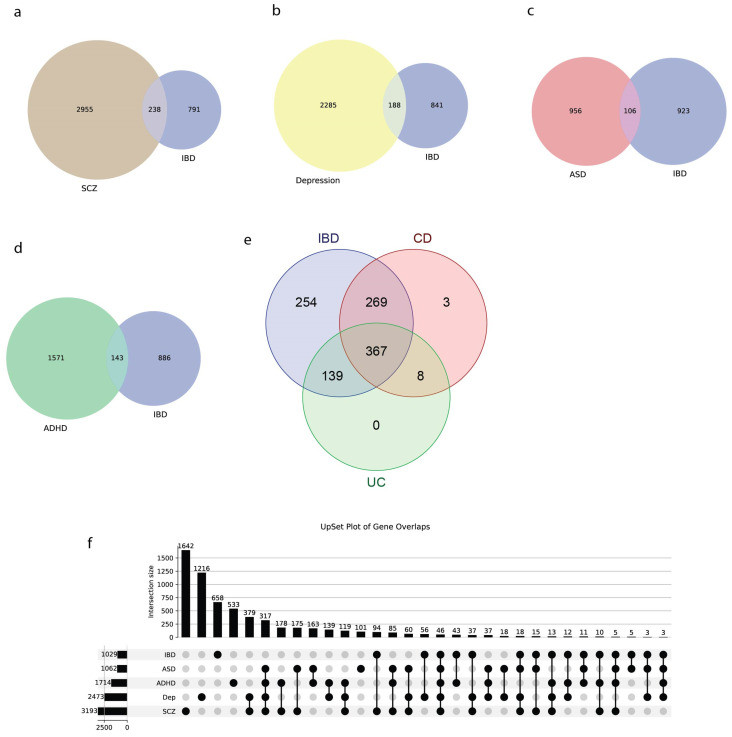
Genetic Overlap Between IBD and Brain Disorders (**a**–**d**) Venn diagrams showing pairwise gene overlaps between IBD and SCZ, Depression, ASD, and ADHD. (**e**) Three-way Venn of IBD, CD, and UC genes. (**f**) UpSet plot detailing all multi-trait intersections, highlighting, for example, 72 genes shared by IBD and ADHD.

We first focused on the genetic overlap among IBD, CD, and UC. We identified a total of 1040 unique genes across IBD, CD, and UC datasets. Among them, 367 genes were common to all three diseases, 269 shared between IBD and CD, and 139 between IBD and UC, with minimal disease-specific unique genes [31], as shown in Figure 5e.

Venn diagrams in Figure 5a–d display the number of shared genes between IBD and each neuropsychiatric disorder, while Supplementary Figure S1 provides the statistical metrics—including overlap odds ratios (ORs) and FDR-adjusted *p*-values—underpinning these overlaps. IBD showed significant genetic overlap with autism spectrum disorder (ASD), which had the strongest enrichment among tested conditions (106 genes; OR = 2.16, 95% CI: 1.73–2.68; adjusted *p* = 4.38 × 10^−11^; enrichment ratio = 1.94; Figure 5c and Figure S1b,c). IBD also overlapped with ADHD, involving 143 shared genes and showing significant enrichment (OR = 1.79, 95% CI: 1.48–2.15; adjusted *p* = 3.98 × 10^−9^; enrichment ratio = 1.62; Figure 5d and Figure S1b,c).

IBD showed significant genetic overlap with Depression (188 genes, OR = 1.63, 95% CI: 1.38–1.93, adjusted *p* = 1.37 × 10^−8^, enrichment ratio = 1.48, Figure 5b and Figure S1b,c). The observed overlap was 48% higher than expected under the null hypothesis. Similarly, significant overlap was observed between IBD and schizophrenia (238 genes, OR = 1.63, 95% CI: 1.40–1.90, adjusted *p* = 6.52 × 10^−10^, enrichment ratio = 1.45, Figure 5a and Figure S1b,c), suggesting shared involvement of immune system pathways in schizophrenia pathogenesis. This represents a 45% enrichment over the expected overlap of 164 genes.

An UpSet plot (Figure 5f) provides a detailed visualization of gene set intersections among all conditions. Notably, the comprehensive pairwise comparison matrix (Supplementary Figure S1d) revealed additional significant overlaps among brain disorders themselves, providing important context for our findings. The strongest overlap was observed between ASD and ADHD (667 genes, OR = 28.82, 95% CI: 25.06–33.25, adjusted *p* < 2.2 × 10^−16^, enrichment ratio = 7.33), followed by SCZ and ASD (721 genes, OR = 14.08, 95% CI: 12.28–16.17) and Depression and ASD (502 genes, OR = 7.71, 95% CI: 6.77–8.80). These substantial overlaps among neuropsychiatric conditions demonstrate the robust shared genetic architecture within the brain disorder spectrum.

All IBD-brain disorder comparisons demonstrated statistically significant genetic overlaps after multiple testing correction (all adjusted *p* < 0.05), with effect sizes (odds ratios) ranging from 1.63 to 2.16. Importantly, the enrichment ratios (1.45–1.94) indicate that the observed overlaps are 45–94% higher than expected by chance alone, providing strong statistical evidence for shared genetic susceptibility factors between IBD and neuropsychiatric conditions. These findings support the growing evidence for the gut–brain axis and suggest common inflammatory and immune-mediated pathways underlying both gastrointestinal and neurological disorders.

## 4. Discussion

The present study provides a comprehensive analysis of the genetic landscape of IBD, focusing on its two main subtypes, CD and UC. By leveraging genome-wide association studies (GWAS) data and integrating findings from the Genotype-Tissue Expression (GTEx) project, we have explored the expression patterns of IBD-associated genes across various tissues, particularly the GIT and the brain. Our analyses highlight the systemic nature of IBD and its potential links to neurological and psychiatric disorders.

### 4.1. Genetic Insights and Pathway Enrichment

Our study identified several essential genes associated with IBD, CD, and UC, with notable overlaps among these conditions. Genes such as *NOD2*, *IL23R*, and *STAT3* were consistently highlighted across multiple analyses, underscoring their critical roles in IBD pathogenesis. The pathway enrichment analyses revealed significant involvement of immune-related pathways, including the Inflammatory Bowel Disease Pathway, Th17 Cell Differentiation, NF-Kappa B Signaling Pathway, and Cytokine-Cytokine Receptor Interaction. These pathways are well-known for their roles in mediating immune and inflammatory responses, which are central to the pathology of IBD [11,32]. Overall, there were 72 common Kegg pathways among IBD, CD, and UC, which were enriched with IBD genes. Also, there were seven unique pathways to IBD, ten unique pathways to UC, and two pathways unique to CD.

The enrichment of the HIF-1 Signaling Pathway in both CD and UC, as well as the broader IBD category, highlights the role of hypoxia in modulating inflammatory responses and tissue remodeling in the gut [33]. The JAK-STAT Signaling Pathway, significantly enriched in both CD and UC, is crucial for transducing signals from cytokines and growth factors, influencing gene expression and immune cell function [34,35].

### 4.2. Genetic Architecture and SNP Distribution in IBD

Our analysis of 69 genome-wide significant SNPs across the 26 shared IBD genes revealed a nuanced balance of common and subtype-specific risk variants: 49.3% were shared between UC and CD, while the remainder split nearly evenly between UC-only and CD-only signals. This pattern both reinforces the clinical continuum of IBD and underscores distinct molecular pathways driving each subtype.

*IL23R* stands out as a paradigm of this complexity, harboring seven risk alleles with divergent associations—three UC-only, one CD-only, and three shared—highlighting its central role in IL-23/IL-17 signaling and suggesting that different molecular perturbations at the same locus may underlie subtype-specific inflammation. *ATG16L1*′s predominance of CD-specific variants further accentuates autophagy dysfunction as a CD hallmark.

The near-equal split of shared versus specific variants may suggest that while broad immune-regulatory mechanisms (for example, NF-κB and JAK-STAT pathways) are common therapeutic targets, tailored interventions addressing subtype-unique variants (e.g., autophagy modulators in CD) may improve precision medicine strategies. Moreover, the chromosomal clustering of UC-specific SNPs on chromosomes 1 and 6 versus CD loci on 10 and 16 hints at region-focused regulatory architectures that warrant deeper functional dissection.

Finally, the polygenic burden across multiple loci—beyond *IL23R* and *ATG16L1*, genes such as *CARD9*, *STAT3* and *PTPN2* each contribute multiple SNPs—confirms IBD’s multifactorial nature and argues against single-gene screening approaches. Instead, comprehensive genotype profiling will be essential for accurate risk stratification and for guiding pathway-based therapeutics. Mapping variants onto the genome uncovers distinct hotspots: UC-only SNPs concentrate on chromosomes 1 and 6, CD-only on 10 and 16, and shared variants on 7 and 19 (Supplementary Figure S2). These clusters suggest region-specific regulatory elements that merit targeted functional assays (e.g., CRISPR-based enhancer screens). Subtype-specific chromosomal hotspots (chr. 1,6 for UC; chr. 10,16 for CD; and chr. 7,19 shared) further illustrate distinct yet convergent genetic architectures underlying IBD [4,6].

### 4.3. Expression Patterns Along the Gut–Brain Axis

Integrating GTEx data allowed us to assess the baseline expression of IBD-associated genes across the gut–brain axis. As expected, these genes were highly expressed in GIT tissues, but they also showed substantial expression in brain regions, particularly the cerebellum, frontal cortex, and hippocampus. Notably, expression patterns were relatively uniform across GIT regions, yet displayed striking heterogeneity across brain areas, indicating potential region-specific neurological roles. Several genes illustrate this dual involvement: CAMK2A, a synaptic plasticity regulator, and PARK7 (DJ-1), a neuroprotective oxidative stress–related gene, were among the most highly expressed in brain tissue, while HLA-DRA was prominently expressed in both gut and brain, consistent with shared immune-mediated mechanisms. Several enriched pathways support the neurobiological relevance of IBD-associated genes.

Although our GTEx analyses characterize baseline (non-diseased) tissue expression, multiple studies document disease-state dysregulation of the same axes in patient tissues. In colonic mucosa from IBD, components of the IL-23/Th17 pathway are elevated and IL23R is increased in patient samples relative to controls [36,37]. Moreover, IL23R^+^ intestinal T-cell populations expand in Crohn’s disease and associate with anti-TNF non-response [38]. In neurological disorders, large postmortem cortex meta-analyses report immune/microglial module up-regulation and JAK–STAT/cytokine signatures across schizophrenia, autism spectrum disorder, and related conditions [39,40,41,42]. Together, these patient-tissue data support the biological plausibility that IBD genetic risk intersects with disease-state immune processes in gut and brain, even though GTEx itself is limited to non-diseased donors.

The neurotrophin signaling pathway is essential for neuronal survival and synaptic plasticity [43]. The Oxytocin signaling pathway, involved in social behavior and emotional regulation, may have relevance for psychiatric comorbidity in IBD [44,45]. In addition, Calcium and Sphingolipid signaling pathways play critical roles in neurotransmission and neuroinflammatory processes [46,47]. The enrichment of Notch and Wnt signaling pathways suggests additional links between IBD genes, immune regulation, and neurodevelopment [48,49,50,51]. Collectively, these pathways highlight mechanistic avenues through which gut inflammation and genetic risk may converge on neural circuits, reinforcing the systemic nature of IBD.

These findings extend existing perspectives by highlighting region-specific expression heterogeneity, supporting the recognition of IBD as a systemic inflammatory condition with neurological implications. The enrichment of brain-related pathways, such as neurotrophin signaling and neurodegeneration pathways, highlight possible mechanistic links to psychiatric comorbidities. This is consistent with clinical observations of increased prevalence of disorders such as schizophrenia, depression, and ASD in IBD cohorts [24,52,53]. Collectively, these results suggest that IBD susceptibility genes may contribute to both intestinal inflammation and extra-intestinal manifestations through regionally specialized roles in the brain.

### 4.4. Genetic Overlap with Brain Disorders

The significant genetic overlap between IBD and neuropsychiatric disorders provides the first comprehensive molecular evidence for the gut–brain axis in inflammatory disease. The strongest overlap with autism spectrum disorder (nearly 2-fold enrichment) is particularly noteworthy, as it may suggests shared neurodevelopmental and immune regulatory mechanisms that may explain the increased prevalence of ASD in families with IBD [54,55,56,57]. Epidemiological studies also report bidirectional risk, with psychiatric patients showing increased incidence of IBD and vice versa [24,27], consistent with our observed genetic overlaps.

These findings have profound implications for clinical practice, suggesting that IBD patients may benefit from screening for neuropsychiatric symptoms and that integrated treatment approaches addressing both gastrointestinal and neurological symptoms may be more effective than traditional organ-specific therapies.

### 4.5. Therapeutic and Repurposing Implications Across the Gut-Brain Axis

Our analyses highlight several pathways with direct translational relevance for therapy. The IL-23/Th17 axis (*IL23R*, *STAT3*) is already targeted in IBD by monoclonal antibodies such as ustekinumab (IL-12/23p40) and risankizumab (IL-23p19), initially developed for psoriasis and later repurposed for Crohn’s disease and ulcerative colitis [58,59]. Similarly, the enrichment of JAK–STAT signaling supports the therapeutic use of JAK inhibitors, such as tofacitinib and upadacitinib, approved in ulcerative colitis [34,60,61]. The prominence of TNF/NF-κB signaling aligns with the efficacy of anti-TNF agents like infliximab and adalimumab [10], while autophagy genes such as *ATG16L1* point to novel opportunities for therapies that modulate cellular homeostasis [62].

Beyond IBD, our findings of genetic overlap with neuropsychiatric disorders suggest repurposing opportunities across the gut–brain axis. Anti-TNF agents, validated in IBD, are being investigated for treatment-resistant depression, with infliximab showing benefit in patients with elevated inflammatory markers [63,64]. JAK inhibitors such as tofacitinib (approved for ulcerative colitis) and baricitinib exhibit neuroprotective effects in experimental models of neuroinflammation. While preclinical studies support their potential in multiple sclerosis, baricitinib is currently being clinically investigated in Alzheimer’s disease and related neurodegenerative conditions [65,66]. The IL-23/IL-17 axis, enriched in our dataset, has also been implicated in neuropsychiatric disease [67,68,69], with immune dysregulation contributing to autism spectrum disorder [55,70].

Conversely, neurological drugs may hold therapeutic value in IBD. Lithium, a GSK-3β inhibitor and autophagy modulator, has demonstrated pro-repair and anti-inflammatory effects in preclinical gut models, including enhanced mucosal regeneration after DSS colitis and attenuation of experimental colitis via microbiota- and Treg-dependent mechanisms [71,72]. Notably, results are model-dependent, with at least one study reporting no benefit in DSS colitis [73]. Given that *ATG16L1* risk variants impair autophagy in intestinal Paneth cells [74] and lithium modulates autophagy pathways [75,76], genotype-specific responsiveness to lithium therapy represents a testable precision medicine hypothesis that merits targeted evaluation. Acetylcholinesterase inhibitors, particularly donepezil, have demonstrated anti-inflammatory and barrier-protective effects in experimental colitis. Donepezil halted acetic acid–induced colitis in rats by reducing oxidative stress, apoptosis, and inflammatory markers [77], and also ameliorated gut barrier disruption in doxorubicin-treated rats [78]. These findings support the involvement of the cholinergic anti-inflammatory pathway, consistent with our enrichment of cholinergic synapse pathways. In parallel, neurotrophin modulation has been implicated in gut–brain regulation: brain-derived neurotrophic factor (BDNF) reduced epithelial apoptosis and preserved tight junction proteins in experimental colitis, thereby protecting barrier integrity [79,80]. Dysbiosis-related changes in BDNF expression further link intestinal and neuropsychiatric pathology [81]. While human validation is lacking, these mechanistic insights suggest that cholinergic and neurotrophin-based therapies may hold dual benefit for intestinal inflammation and comorbid psychiatric manifestations in IBD.

Finally, our results support a precision repurpose framework. Patients with IBD carrying high polygenic risk scores for neuropsychiatric disorders may benefit from gut-directed therapies with favorable CNS profiles, while psychiatric patients with elevated IBD genetic risk might benefit from anti-inflammatory augmentation. This bidirectional, genetically guided strategy represents a cost-effective approach to addressing the complex comorbidities spanning IBD and neuropsychiatric disease.

### 4.6. Strengths and Limitations

One of the strengths of this study is the integration of large-scale GWAS data with comprehensive expression datasets from the GTEx project. This approach provides a robust framework for identifying key genes and pathways involved in IBD and exploring their broader systemic effects. As GTEx contains expression data only from non-diseased tissues, our analysis reflects baseline rather than disease-state expression patterns. While RNA-seq datasets from patient biopsies (e.g., GEO) exist, they are typically limited to specific gastrointestinal tissues and rarely have corresponding neurological samples. We therefore used GTEx to map tissue-specific expression as a proxy for identifying which brain regions may be most influenced by IBD genetic background, while acknowledging that this approach does not capture disease-specific dysregulation. Additionally, the use of multiple visualization techniques, including Venn diagrams, enhances interpretability of complex gene–pathway–disease relationships.

However, there are some limitations to consider. Our cross-sectional GTEx analysis cannot capture dynamic expression during disease flares or treatment, and the predominance of European-ancestry GWAS data may limit generalizability. Moreover, transcript-level associations do not necessarily imply variant functionality at the protein level. Beyond baseline GTEx expression, disease-state validation can leverage public biopsy and postmortem resources—for example, IBD colon mucosa cohorts profiled by Arijs and colleagues [82] and cross-disorder brain transcriptomics [39]. A harmonized, cross-platform meta-analysis spanning cohorts and normalization procedures would be valuable but is beyond the scope of the present synthesis.

### 4.7. Future Directions

Future research should aim to validate these findings using longitudinal data and explore the functional roles of identified genes and pathways in detail. While our analysis focused on gene-level associations and expression patterns, future work integrating colocalization, eQTL mapping, and chromatin interaction data will be essential to establish causal links between IBD and neuropsychiatric traits. Integrating additional omics data, such as proteomics and metabolomics, could provide deeper insights into the molecular mechanisms underlying IBD and its associated comorbidities. Furthermore, investigating the impact of environmental factors, such as diet and microbiota, on gene expression and disease progression could help elucidate the complex interplay between genetic and environmental determinants of IBD.

Induced pluripotent stem cells (iPSCs) have revolutionized the study of various neurological disorders, such as Autism Spectrum Disorder, Schizophrenia, bipolar disorder, Parkinson’s disease, and more [83,84,85,86,87,88,89,90] by enabling researchers to create patient-specific neurons and brain organoids. These organoids provide an invaluable platform for modeling complex brain functions and behaviors as well as the underlying pathology of diseases. Researchers can explore the genetic, molecular, and cellular mechanisms implicated in various neurological conditions by converting somatic cells from patients into iPSCs and subsequently differentiating them into neural cell types.

Given the shared signaling pathways and cellular interactions between the gut and the central nervous system, integrating iPSC technology into the gut–brain axis research area presents exciting future directions. One promising avenue involves the development of joint gut–brain organoids derived from iPSCs. These organoids would allow researchers to elucidate the interactions between gut microbiota, the enteric nervous system, and central nervous system neurons in the context of IBD. By studying these organoids, scientists could investigate how inflammatory signals from the gut affect neuronal function and vice versa. Such experiments could lead to a deeper understanding of the mechanisms underlying IBD and its associated neurological comorbidities, such as anxiety and depression, often observed in patients with chronic gastrointestinal disorders.

Furthermore, joint gut–brain organoids could be utilized to screen potential therapeutic compounds that simultaneously target both systems, opening new avenues for integrated treatments that address IBD and its neurological impacts. Ultimately, this innovative approach may provide insights into the interconnectedness of these systems, paving the way for more holistic treatment strategies that recognize the importance of the brain–gut axis in IBD management.

## 5. Conclusions

This work systematically integrates genome-wide association data with tissue-specific expression profiles to provide a comprehensive synthesis of the genetic architecture underlying inflammatory bowel disease (IBD) and its systemic connections with neurological conditions. Re-analysis of public datasets consistently highlights immune-regulatory pathways—such as IL-23/Th17, NF-κB, and cytokine signaling—as central features across IBD subtypes, reaffirming their pivotal role in disease pathogenesis. IBD-associated genes show uniform expression across gastrointestinal tissues but heterogeneous patterns across brain regions, suggesting that the genetic background of IBD may modulate neurological processes and help explain the high burden of comorbid depression, schizophrenia, ADHD, and autism spectrum disorder. Rather than proposing new mechanisms, the present synthesis consolidates existing genomic and transcriptomic findings into an integrative framework that underscores IBD’s multisystem nature. These insights can serve as a reference for future mechanistic, clinical, and translational studies aimed at linking gut and brain pathophysiology. While our analyses rely on baseline (non-diseased) datasets and require functional validation in disease-specific models, they establish a transparent resource for guiding hypothesis generation and study design—particularly for emerging approaches using patient-derived systems such as iPSC-based gut–brain organoids. By positioning IBD within a broader genetic and molecular context that encompasses both intestinal and neural domains, this synthesis promotes a more integrated understanding of pathogenesis and provides a reference point for precision-medicine strategies addressing the gastrointestinal and neurological dimensions of the disorder.

## Data Availability

Correspondence and requests for code should be addressed to Prof. Shani Stern.

## Supplementary Materials

The following supporting information can be downloaded at: https://www.mdpi.com/article/10.3390/biology14101433/s1, Figure S1: Genetic overlap analysis between IBD and neurodevelopmental/psychiatric disorders. (**a**) GWAS Dataset Summary. A bar chart showing the number of unique genes and studies per condition from GWAS Catalog downloads (2024). IBD: Inflammatory Bowel Disease (1029 genes, 87 studies); ASD: Autism Spectrum Disorder (1062 genes, 28 studies); ADHD: Attention Deficit Hyperactivity Disorder (1714 genes, 43 studies); Depression (2473 genes, 97 studies); SCZ: Schizophrenia (3193 genes, 103 studies). (**b**) Genetic Overlap Enrichment Analysis. Enrichment ratios (observed/expected) for genetic overlap between IBD and neurodevelopmental/psychiatric disorders. Numbers above bars indicate the count of overlapping genes. The dashed line indicates no enrichment (ratio = 1). All comparisons showed significant enrichment: ADHD (143 genes, *p* < 0.001), ASD 106 genes, *p* < 0.001), Depression (188 genes, *p* < 0.001), SCZ (238 genes, *p* < 0.001). Statistical significance: *** *p* < 0.001, ** *p* < 0.01, * *p* < 0.05, ns = not significant. (**c**) Odds Ratios for Genetic Overlap. A forest plot showing odds ratios (OR) with 95% confidence intervals for genetic overlap between IBD and brain disorders. Y-axis is presented on log scale. The dashed line indicates no association (OR = 1). All disorders showed significant positive association with IBD: ADHD (OR = 1.79, *n* = 143), ASD (OR = 2.16, *n* = 106), Depression (OR = 1.63, *n* = 188), SCZ (OR = 1.63, *n* = 238). Statistical significance: *** *p* < 0.001, ** *p* < 0.01, * *p* < 0.05, ns = not significant. (**d**) Pairwise Genetic Overlap Significance Matrix. A heatmap showing pairwise comparisons between all conditions (IBD, ADHD, ASD, Depression, SCZ). Color intensity represents -log10(adjusted *p*-value), with darker colors indicating higher statistical significance. Numbers within cells show odds ratio and gene overlap count (*n*). The matrix demonstrates significant genetic overlap between IBD and all tested neurodevelopmental/psychiatric disorders, with the strongest associations observed for ADHD-ASD (OR = 28.82, *n* = 667) and ASD-SCZ (OR = 14.08, *n* = 721). Figure S2: Chromosomal distribution of IBD-associated genetic variants. (**a**) IBD Genetic Variants Distribution. Circos plot showing the chromosomal distribution of all IBD-associated genetic variants (*n* = 154) across chromosomes 1–22. Variants are color-coded by statistical significance: genome-wide significant (*p* < 1 × 10^−8^), suggestive (1 × 10^−8^ ≤ *p* < 1 × 10^−5^), and nominal (*p* ≥ 1 × 10^−5^). Chromosomes are arranged circularly. Genetic variants are plotted based on their genomic position along each chromosome, with their statistical significance indicated by color. (**b**) Crohn’s Disease SNPs Distribution. Chromosomal distribution of Crohn’s disease-specific single nucleotide polymorphisms (SNPs) (*n* = 88) across all chromosomes. The plot shows the genomic locations of CD-associated variants with the same significance thresholds as panel A. (**c**) Ulcerative Colitis SNPs Distribution. Chromosomal distribution of ulcerative colitis-specific SNPs (*n* = 66) across all chromosomes. The plot displays the genomic locations of UC-associated variants, demonstrating the distinct but overlapping genetic architecture between the two main IBD subtypes. All genetic variants were obtained from GWAS studies and represent established disease associations. The distribution patterns reveal both shared and distinct genetic loci contributing to IBD pathogenesis. Figure S3: Differential expression patterns of IBD-associated genes in brain and gastrointestinal tissues. (**a**) Mean expression in brain regions. Bar chart showing mean expression levels (TPM) of 413 IBD-associated genes across 13 brain regions from GTEx data. Brain regions are ranked by expression level, with cerebellar hemisphere (19.1 TPM) and cerebellum (18.7 TPM) showing the highest expression levels. One-way ANOVA revealed significant differences between brain regions (*p* = 6.37 × 10^−9^). Numbers above bars indicate mean TPM values. (**b**) Mean expression in gastrointestinal (GIT) regions. Bar chart displaying mean expression levels of IBD genes across 7 GIT regions. Esophagus muscularis (29.1 TPM) and esophagus gastroesophageal junction (28.8 TPM) exhibited the highest expression levels. One-way ANOVA showed no significant differences between GIT regions (*p* = 0.339). Numbers above bars indicate mean TPM values. (**c**) Brain versus GIT comparison. Direct comparison of mean IBD gene expression between brain (12.8 TPM) and GIT tissues (22.5 TPM). Mann–Whitney U test revealed significantly higher expression in GIT compared to brain tissues (*p* = 2.04 × 10^−4^). Numbers above bars indicate mean TPM values. (**d**) Top genes expression heatmap. Heatmap showing expression levels of the most highly expressed IBD genes in brain and GIT tissues. The top 10 genes from each tissue type are displayed, with *ACTA2* showing the highest expression in GIT (1446.4 TPM) and *CAMK2A* showing the highest expression in brain (214.4 TPM). Color intensity represents expression level (TPM), with darker red indicating higher expression. Expression data were analyzed using GTEx v8 RNA-seq data (*n* = 56,200 total genes, 413 IBD genes, 55,787 non-IBD genes). IBD genes showed significantly higher expression compared to non-IBD genes in brain (*p* = 9.39 × 10^−82^), GIT (*p* = 2.65 × 10^−97^), and other tissues (*p* = 1.17 × 10^−90^). Kruskal–Wallis test confirmed significant expression differences across tissue groups for both IBD (*p* = 2.55 × 10^−7^) and non-IBD genes (*p* < 2.2 × 10^−16^). Supplementary File S1. Raw GWAS data of IBD-Associated SNPs. This table lists all single-nucleotide polymorphisms (SNPs) cataloged as genome-wide significant for Inflammatory Bowel Disease (IBD), drawn from the NHGRI-EBI GWAS Catalog.

## References

[1] Franke A., McGovern D.P.B., Barrett J.C., Wang K., Radford-Smith G.L., Ahmad T., Lees C.W., Balschun T., Lee J., Roberts R. (2010). Genome-Wide Meta-Analysis Increases to 71 the Number of Confirmed Crohn’s Disease Susceptibility Loci. Nat. Genet..

[2] Jostins L., Ripke S., Weersma R.K., Duerr R.H., McGovern D.P., Hui K.Y., Lee J.C., Philip Schumm L., Sharma Y., The International IBD Genetics Consortium (IIBDGC) (2012). Host–Microbe Interactions Have Shaped the Genetic Architecture of Inflammatory Bowel Disease. Nature.

[3] Xavier R.J., Podolsky D.K. (2007). Unravelling the Pathogenesis of Inflammatory Bowel Disease. Nature.

[4] Liu J.Z., Van Sommeren S., Huang H., Ng S.C., Alberts R., Takahashi A., Ripke S., Lee J.C., Jostins L., Shah T. (2015). Association Analyses Identify 38 Susceptibility Loci for Inflammatory Bowel Disease and Highlight Shared Genetic Risk Across Populations. Nat. Genet..

[5] de Lange K.M., Moutsianas L., Lee J.C., Lamb C.A., Luo Y., Kennedy N.A., Jostins L., Rice D.L., Gutierrez-Achury J., Ji S.-G. (2017). Genome-Wide Association Study Implicates Immune Activation of Multiple Integrin Genes in Inflammatory Bowel Disease. Nat. Genet..

[6] Huang H., Fang M., Jostins L., Umićević Mirkov M., Boucher G., Anderson C.A., Andersen V., Cleynen I., Cortes A., Crins F. (2017). Fine-Mapping Inflammatory Bowel Disease Loci to Single-Variant Resolution. Nature.

[7] Liu H., Guan L., Su X., Zhao L., Shu Q., Zhang J. (2024). A Broken Network of Susceptibility Genes in the Monocytes of Crohn’s Disease Patients. Life Sci. Alliance.

[8] Zhang M., Zhao J., Ji H., Tan Y., Zhou S., Sun J., Ding Y., Li X. (2025). Multi-Omics Insight into the Molecular Networks of Mental Disorder Related Genetic Pathways in the Pathogenesis of Inflammatory Bowel Disease. Transl. Psychiatry.

[9] Carithers L.J., Moore H.M. (2015). The Genotype-Tissue Expression (GTEx) Project. Biopreservation Biobanking.

[10] Danese S., Fiocchi C. (2011). Ulcerative Colitis. N. Engl. J. Med..

[11] Baumgart D.C., Carding S.R. (2007). Inflammatory Bowel Disease: Cause and Immunobiology. Lancet.

[12] Lonsdale J., Thomas J., Salvatore M., Phillips R., Lo E., Shad S., Hasz R., Walters G., Garcia F., Young N. (2013). The Genotype-Tissue Expression (GTEx) Project. Nat. Genet..

[13] Bycroft C., Freeman C., Petkova D., Band G., Elliott L.T., Sharp K., Motyer A., Vukcevic D., Delaneau O., O’Connell J. (2018). The UK Biobank Resource with Deep Phenotyping and Genomic Data. Nature.

[14] Gong W., Guo P., Li Y., Liu L., Yan R., Liu S., Wang S., Xue F., Zhou X., Yuan Z. (2023). Role of the Gut-Brain Axis in the Shared Genetic Etiology Between Gastrointestinal Tract Diseases and Psychiatric Disorders: A Genome-Wide Pleiotropic Analysis. JAMA Psychiatry.

[15] Watanabe K., Taskesen E., van Bochoven A., Posthuma D. (2017). Functional Mapping and Annotation of Genetic Associations with FUMA. Nat. Commun..

[16] MAGMA: Generalized Gene-Set Analysis of GWAS Data|PLOS Computational Biology. https://journals.plos.org/ploscompbiol/article?id=10.1371/journal.pcbi.1004219.

[17] The GTEx Consortium (2020). The GTEx Consortium Atlas of Genetic Regulatory Effects Across Human Tissues. Science.

[18] Cryan J.F., Dinan T.G. (2012). Mind-Altering Microorganisms: The Impact of the Gut Microbiota on Brain and Behaviour. Nat. Rev. Neurosci..

[19] Wachsmuth H.R., Weninger S.N., Duca F.A. (2022). Role of the Gut–Brain Axis in Energy and Glucose Metabolism. Exp. Mol. Med..

[20] La Torre D., Van Oudenhove L., Vanuytsel T., Verbeke K. (2023). Psychosocial Stress-Induced Intestinal Permeability in Healthy Humans: What Is the Evidence?. Neurobiol. Stress.

[21] Benros M.E., Waltoft B.L., Nordentoft M., Østergaard S.D., Eaton W.W., Krogh J., Mortensen P.B. (2013). Autoimmune Diseases and Severe Infections as Risk Factors for Mood Disorders: A Nationwide Study. JAMA Psychiatry.

[22] Mikocka-Walus A.A., Turnbull D.A., Moulding N.T., Wilson I.G., Andrews J.M., Holtmann G.J. (2007). Controversies Surrounding the Comorbidity of Depression and Anxiety in Inflammatory Bowel Disease Patients: A Literature Review. Inflamm. Bowel Dis..

[23] Bhamre R., Sawrav S., Adarkar S., Sakaria R., Bhatia S.J. (2018). Psychiatric Comorbidities in Patients with Inflammatory Bowel Disease. Indian J. Gastroenterol..

[24] Bernstein C.N., Hitchon C.A., Walld R., Bolton J.M., Sareen J., Walker J.R., Graff L.A., Patten S.B., Singer A., Lix L.M. (2019). Increased Burden of Psychiatric Disorders in Inflammatory Bowel Disease. Inflamm. Bowel Dis..

[25] Wang J., Luo G.-Y., Tian T., Zhao Y.-Q., Meng S.-Y., Wu J.-H., Han W.-X., Deng B., Ni J. (2024). Shared Genetic Basis and Causality between Schizophrenia and Inflammatory Bowel Disease: Evidence from a Comprehensive Genetic Analysis. Psychol. Med..

[26] Ge L., Liu S., Li S., Yang J., Hu G., Xu C., Song W. (2022). Psychological Stress in Inflammatory Bowel Disease: Psychoneuroimmunological Insights into Bidirectional Gut–Brain Communications. Front. Immunol..

[27] Sung K., Zhang B., Wang H.E., Bai Y., Tsai S., Su T., Chen T., Hou M., Lu C., Wang Y. (2022). Schizophrenia and Risk of New-onset Inflammatory Bowel Disease: A Nationwide Longitudinal Study. Aliment. Pharmacol. Ther..

[28] Choudhary A., Nayak R., Peles D., Mizrahi L., Stern S. (2022). Current Progress in Understanding Schizophrenia Using Genomics and Pluripotent Stem Cells: A Meta-Analytical Overview. Schizophr. Res..

[29] Romanovsky E., Choudhary A., Peles D., Akel A.A., Stern S. (2023). Uncovering Convergence and Divergence between Autism and Schizophrenia Using Genomic Tools and Patients’ Neurons. Mol. Psychiatry.

[30] Fakhfouri G., Mijailović N.R., Rahimian R. (2024). Psychiatric Comorbidities of Inflammatory Bowel Disease: It Is a Matter of Microglia’s Gut Feeling. Cells.

[31] Waterman M., Xu W., Stempak J.M., Milgrom R., Bernstein C.N., Griffiths A.M., Greenberg G.R., Steinhart H.A., Silverberg M.S. (2011). Distinct and Overlapping Genetic Loci in Crohn’s Disease and Ulcerative Colitis: Correlations with Pathogenesis. Inflamm. Bowel Dis..

[32] Atreya I., Atreya R., Neurath M.F. (2008). NF-κB in Inflammatory Bowel Disease. J. Intern. Med..

[33] Kerber E.L., Padberg C., Koll N., Schuetzhold V., Fandrey J., Winning S. (2020). The Importance of Hypoxia-Inducible Factors (HIF-1 and HIF-2) for the Pathophysiology of Inflammatory Bowel Disease. Int. J. Mol. Sci..

[34] Coskun M., Salem M., Pedersen J., Nielsen O.H. (2013). Involvement of JAK/STAT Signaling in the Pathogenesis of Inflammatory Bowel Disease. Pharmacol. Res..

[35] Schindler C., Levy D.E., Decker T. (2007). JAK-STAT Signaling: From Interferons to Cytokines. J. Biol. Chem..

[36] Kobayashi T., Okamoto S., Hisamatsu T., Kamada N., Chinen H., Saito R., Kitazume M.T., Nakazawa A., Sugita A., Koganei K. (2008). IL23 Differentially Regulates the Th1/Th17 Balance in Ulcerative Colitis and Crohn’s Disease. Gut.

[37] Song L., Zhou R., Huang S., Zhou F., Xu S., Wang W., Yi F., Wang X., Xia B. (2013). High Intestinal and Systemic Levels of Interleukin-23/T-Helper 17 Pathway in Chinese Patients with Inflammatory Bowel Disease. Mediat. Inflamm..

[38] Schmitt H., Billmeier U., Dieterich W., Rath T., Sonnewald S., Reid S., Hirschmann S., Hildner K., Waldner M.J., Mudter J. (2019). Expansion of IL-23 Receptor Bearing TNFR2+ T Cells Is Associated with Molecular Resistance to Anti-TNF Therapy in Crohn’s Disease. Gut.

[39] Gandal M.J., Haney J.R., Parikshak N.N., Leppa V., Ramaswami G., Hartl C., Schork A.J., Appadurai V., Buil A., Werge T.M. (2018). Shared Molecular Neuropathology across Major Psychiatric Disorders Parallels Polygenic Overlap. Science.

[40] Lanz T.A., Reinhart V., Sheehan M.J., Rizzo S.J.S., Bove S.E., James L.C., Volfson D., Lewis D.A., Kleiman R.J. (2019). Postmortem Transcriptional Profiling Reveals Widespread Increase in Inflammation in Schizophrenia: A Comparison of Prefrontal Cortex, Striatum, and Hippocampus among Matched Tetrads of Controls with Subjects Diagnosed with Schizophrenia, Bipolar or Major Depressive Disorder. Transl. Psychiatry.

[41] Voineagu I., Wang X., Johnston P., Lowe J.K., Tian Y., Horvath S., Mill J., Cantor R.M., Blencowe B.J., Geschwind D.H. (2011). Transcriptomic Analysis of Autistic Brain Reveals Convergent Molecular Pathology. Nature.

[42] Lindholm Carlström E., Niazi A., Etemadikhah M., Halvardson J., Enroth S., Stockmeier C.A., Rajkowska G., Nilsson B., Feuk L. (2021). Transcriptome Analysis of Post-Mortem Brain Tissue Reveals Up-Regulation of the Complement Cascade in a Subgroup of Schizophrenia Patients. Genes.

[43] Huang E.J., Reichardt L.F. (2001). Neurotrophins: Roles in Neuronal Development and Function. Annu. Rev. Neurosci..

[44] Jin Y., Song D., Yan Y., Quan Z., Qing H. (2023). The Role of Oxytocin in Early-Life-Stress-Related Neuropsychiatric Disorders. Int. J. Mol. Sci..

[45] Zois C.D., Katsanos K.H., Kosmidou M., Tsianos E.V. (2010). Neurologic Manifestations in Inflammatory Bowel Diseases: Current Knowledge and Novel Insights. J. Crohn’s Colitis.

[46] Pulli I., Asghar M.Y., Kemppainen K., Törnquist K. (2018). Sphingolipid-Mediated Calcium Signaling and Its Pathological Effects. Biochim. Biophys. Acta (BBA)-Mol. Cell Res..

[47] Lee J.Y., Jin H.K., Bae J. (2020). Sphingolipids in Neuroinflammation: A Potential Target for Diagnosis and Therapy. BMB Rep..

[48] Fre S., Bardin A., Robine S., Louvard D. (2011). Notch Signaling in Intestinal Homeostasis across Species: The Cases of Drosophila, Zebrafish and the Mouse. Exp. Cell Res..

[49] Lasky J.L., Wu H. (2005). Notch Signaling, Brain Development, and Human Disease. Pediatr. Res..

[50] Inestrosa N.C., Varela-Nallar L. (2014). Wnt Signaling in the Nervous System and in Alzheimer’s Disease. J. Mol. Cell Biol..

[51] Patapoutian A., Reichardt L.F. (2000). Roles of Wnt Proteins in Neural Development and Maintenance. Curr. Opin. Neurobiol..

[52] Benros M.E., Nielsen P.R., Nordentoft M., Eaton W.W., Dalton S.O., Mortensen P.B. (2011). Autoimmune Diseases and Severe Infections as Risk Factors for Schizophrenia: A 30-Year Population-Based Register Study. Am. J. Psychiatry.

[53] Mikocka-Walus A., Pittet V., Rossel J.-B., Von Känel R., Anderegg C., Bauerfeind P., Beglinger C., Begré S., Belli D., Bengoa J.M. (2016). Symptoms of Depression and Anxiety Are Independently Associated with Clinical Recurrence of Inflammatory Bowel Disease. Clin. Gastroenterol. Hepatol..

[54] Masi A., Quintana D.S., Glozier N., Lloyd A.R., Hickie I.B., Guastella A.J. (2015). Cytokine Aberrations in Autism Spectrum Disorder: A Systematic Review and Meta-Analysis. Mol. Psychiatry.

[55] Estes M.L., McAllister A.K. (2015). Immune Mediators in the Brain and Peripheral Tissues in Autism Spectrum Disorder. Nat. Rev. Neurosci..

[56] Zawadzka A., Cieślik M., Adamczyk A. (2021). The Role of Maternal Immune Activation in the Pathogenesis of Autism: A Review of the Evidence, Proposed Mechanisms and Implications for Treatment. Int. J. Mol. Sci..

[57] Kim J.Y., Choi M.J., Ha S., Hwang J., Koyanagi A., Dragioti E., Radua J., Smith L., Jacob L., Salazar de Pablo G. (2022). Association between Autism Spectrum Disorder and Inflammatory Bowel Disease: A Systematic Review and Meta-Analysis. Autism. Res..

[58] Sewell G.W., Kaser A. (2022). Interleukin-23 in the Pathogenesis of Inflammatory Bowel Disease and Implications for Therapeutic Intervention. J. Crohn’s Colitis.

[59] Louis E., Schreiber S., Panaccione R., Bossuyt P., Biedermann L., Colombel J.-F., Parkes G., Peyrin-Biroulet L., D’Haens G., Hisamatsu T. (2024). Risankizumab for Ulcerative Colitis: Two Randomized Clinical Trials. JAMA.

[60] Sandborn W.J., Su C., Sands B.E., D’Haens G.R., Vermeire S., Schreiber S., Danese S., Feagan B.G., Reinisch W., Niezychowski W. (2017). Tofacitinib as Induction and Maintenance Therapy for Ulcerative Colitis. N. Engl. J. Med..

[61] RINVOQ® (Upadacitinib) Receives FDA Approval for the Treatment of Adults with Moderately to Severely Active Ulcerative Colitis. https://news.abbvie.com/2022-03-16-RINVOQ-R-upadacitinib-Receives-FDA-Approval-for-the-Treatment-of-Adults-with-Moderately-to-Severely-Active-Ulcerative-Colitis.

[62] Naser S.A. (2012). Role of *ATG16L*, *NOD2* and *IL23R* in Crohn’s Disease Pathogenesis. World J. Gastroenterol..

[63] Kappelmann N., Lewis G., Dantzer R., Jones P.B., Khandaker G.M. (2018). Antidepressant Activity of Anti-Cytokine Treatment: A Systematic Review and Meta-Analysis of Clinical Trials of Chronic Inflammatory Conditions. Mol. Psychiatry.

[64] Raison C.L., Rutherford R.E., Woolwine B.J., Shuo C., Schettler P., Drake D.F., Haroon E., Miller A.H. (2013). A Randomized Controlled Trial of the Tumor Necrosis Factor Antagonist Infliximab for Treatment-Resistant Depression: The Role of Baseline Inflammatory Biomarkers. JAMA Psychiatry.

[65] Benveniste E.N., Liu Y., McFarland B.C., Qin H. (2014). Involvement of the Janus Kinase/Signal Transducer and Activator of Transcription Signaling Pathway in Multiple Sclerosis and the Animal Model of Experimental Autoimmune Encephalomyelitis. J. Interferon. Cytokine Res..

[66] Faquetti M.L., Slappendel L., Bigonne H., Grisoni F., Schneider P., Aichinger G., Schneider G., Sturla S.J., Burden A.M. (2024). Baricitinib and Tofacitinib Off-target Profile, with a Focus on Alzheimer’s Disease. Alzheimer’s Dement. N. Y..

[67] Ghasemi Noghabi P., Shahini N., Salimi Z., Ghorbani S., Bagheri Y., Derakhshanpour F. (2024). Elevated Serum IL-17 A and CCL20 Levels as Potential Biomarkers in Major Psychotic Disorders: A Case-Control Study. BMC Psychiatry.

[68] Moulton C.D., Malys M., Hopkins C.W.P., Rokakis A.S., Young A.H., Powell N. (2024). Activation of the Interleukin-23/Th17 Axis in Major Depression: A Systematic Review and Meta-Analysis. Eur. Arch. Psychiatry Clin. Neurosci..

[69] Al-Hakeim H.K., Al-Musawi A.F., Al-Mulla A., Al-Dujaili A.H., Debnath M., Maes M. (2022). The Interleukin-6/Interleukin-23/T Helper 17-Axis as a Driver of Neuro-Immune Toxicity in the Major Neurocognitive Psychosis or Deficit Schizophrenia: A Precision Nomothetic Psychiatry Analysis. PLoS ONE.

[70] Hughes H.K., Moreno R.J., Ashwood P. (2023). Innate Immune Dysfunction and Neuroinflammation in Autism Spectrum Disorder (ASD). Brain Behav. Immun..

[71] Raup-Konsavage W.M., Cooper T.K., Yochum G.S. (2016). A Role for MYC in Lithium-Stimulated Repair of the Colonic Epithelium After DSS-Induced Damage in Mice. Dig. Dis. Sci..

[72] Huang S., Hu S., Liu S., Tang B., Liu Y., Tang L., Lei Y., Zhong L., Yang S., He S. (2022). Lithium Carbonate Alleviates Colon Inflammation through Modulating Gut Microbiota and Treg Cells in a GPR43-Dependent Manner. Pharmacol. Res..

[73] van der Logt E.M.J., Blokzijl T., Diepstra A., Peppelenbosch M.P., Huls G., Faber K.N., Dijkstra G. (2012). Lithium Induces Intestinothrophic Effects in the Healthy Colon, but Does Not Ameliorate Dextran Sulfate Sodium-Induced Colitis in Mice. E-SPEN J..

[74] Cadwell K., Liu J.Y., Brown S.L., Miyoshi H., Loh J., Lennerz J.K., Kishi C., Kc W., Carrero J.A., Hunt S. (2008). A Key Role for Autophagy and the Autophagy Gene *ATG16L1* in Mouse and Human Intestinal Paneth Cells. Nature.

[75] Puglisi-Allegra S., Lazzeri G., Busceti C.L., Giorgi F.S., Biagioni F., Fornai F. (2023). Lithium Engages Autophagy for Neuroprotection and Neuroplasticity: Translational Evidence for Therapy. Neurosci. Biobehav. Rev..

[76] Motoi Y., Shimada K., Ishiguro K., Hattori N. (2014). Lithium and Autophagy. ACS Chem. Neurosci..

[77] Elbaz E.M., Essam R.M., Ahmed K.A., Safwat M.H. (2023). Donepezil Halts Acetic Acid-Induced Experimental Colitis in Rats and Its Associated Cognitive Impairment through Regulating Inflammatory/Oxidative/Apoptotic Cascades: An Add-on to Its Anti-Dementia Activity. Int. Immunopharmacol..

[78] Suparan K., Sriwichaiin S., Thonusin C., Sripetchwandee J., Khuanjing T., Maneechote C., Nawara W., Arunsak B., Chattipakorn N., Chattipakorn S.C. (2024). Donepezil Ameliorates Gut Barrier Disruption in Doxorubicin-Treated Rats. Food Chem. Toxicol..

[79] Yu Y.-B., Zhao D.-Y., Qi Q.-Q., Long X., Li X., Chen F.-X., Zuo X.-L. (2017). BDNF Modulates Intestinal Barrier Integrity through Regulating the Expression of Tight Junction Proteins. Neurogastroenterol. Motil..

[80] Zhao D.Y., Zhang W.X., Qi Q.Q., Long X., Li X., Yu Y.B., Zuo X.L. (2018). Brain-Derived Neurotrophic Factor Modulates Intestinal Barrier by Inhibiting Intestinal Epithelial Cells Apoptosis in Mice. Physiol. Res..

[81] Molska M., Mruczyk K., Cisek-Woźniak A., Prokopowicz W., Szydełko P., Jakuszewska Z., Marzec K., Trocholepsza M. (2024). The Influence of Intestinal Microbiota on BDNF Levels. Nutrients.

[82] GEO Accession Viewer. https://www.ncbi.nlm.nih.gov/geo/query/acc.cgi?acc=GSE16879.

[83] Rike W., Stern S. (2023). Proteins and Transcriptional Dysregulation of the Brain Extracellular Matrix in Parkinson’s Disease: A Systematic Review. medRxiv.

[84] Tripathi M.K., Ojha S.K., Kartawy M., Hamoudi W., Choudhary A., Stern S., Aran A., Amal H. (2023). The NO Answer for Autism Spectrum Disorder. Adv. Sci..

[85] Hussein Y., Tripathi U., Choudhary A., Nayak R., Peles D., Rosh I., Djamus J., Spiegel R., Garin-Shkolnik T., Stern S. (2022). Early Maturation and Hyperexcitability Is a Shared Phenotype of Cortical Neurons Derived from Different ASD-Causing Mutations. Transl. Psychiatry.

[86] Stern S., Zhang L., Wang M., Wright R., Rosh I., Hussein Y., Stern T., Choudhary A., Tripathi U., Reed P. (2024). Monozygotic Twins Discordant for Schizophrenia Differ in Maturation and Synaptic Transmission. Mol. Psychiatry.

[87] Figueiredo T., Mendes A.P.D., Moreira D.P., Goulart E., Oliveira D., Kobayashi G.S., Stern S., Kok F., Marchetto M.C., Santos R. (2021). Inositol Monophosphatase 1 (IMPA1) Mutation in Intellectual Disability Patients Impairs Neurogenesis but Not Gliogenesis. Mol. Psychiatry.

[88] Rosh I., Tripathi U.K., Hussein Y., Rike W.A., Djamus J., Shklyar B., Manole A., Houlden H., Winkler J., Gage F.H. (2024). Synaptic Dysfunction and Extracellular Matrix Dysregulation in Dopaminergic Neurons from Sporadic and E326K-*GBA1* Parkinson’s Disease Patients. npj Park. Dis..

[89] Manole A., Wong T., Rhee A., Novak S., Chin S.-M., Tsimring K., Paucar A., Williams A., Newmeyer T.F., Schafer S.T. (2023). NGLY1 Mutations Cause Protein Aggregation in Human Neurons. Cell Rep..

[90] Tripathi U., Rosh I., Ezer R.B., Nayak R., Choudhary A., Djamus J., Manole A., Haulden H., Gage F.H., Stern S. (2023). Upregulated Extracellular Matrix-Related Genes and Impaired Synaptic Activity in Dopaminergic and Hippocampal Neurons Derived from Parkinson’s Disease Patients with PINK1 and PARK2 Mutations. bioRxiv.

