# Supplementary figures and images for: From Genome-Wide SNPs to Neuroimmune Crosstalk: Mapping the Genetic Landscape of IBD and Its Brain Overlap
Source: Biology (Basel). 2025 Oct 17;14(10):1433. doi: 10.3390/biology14101433 (PMC12562180; doi:10.3390/biology14101433)

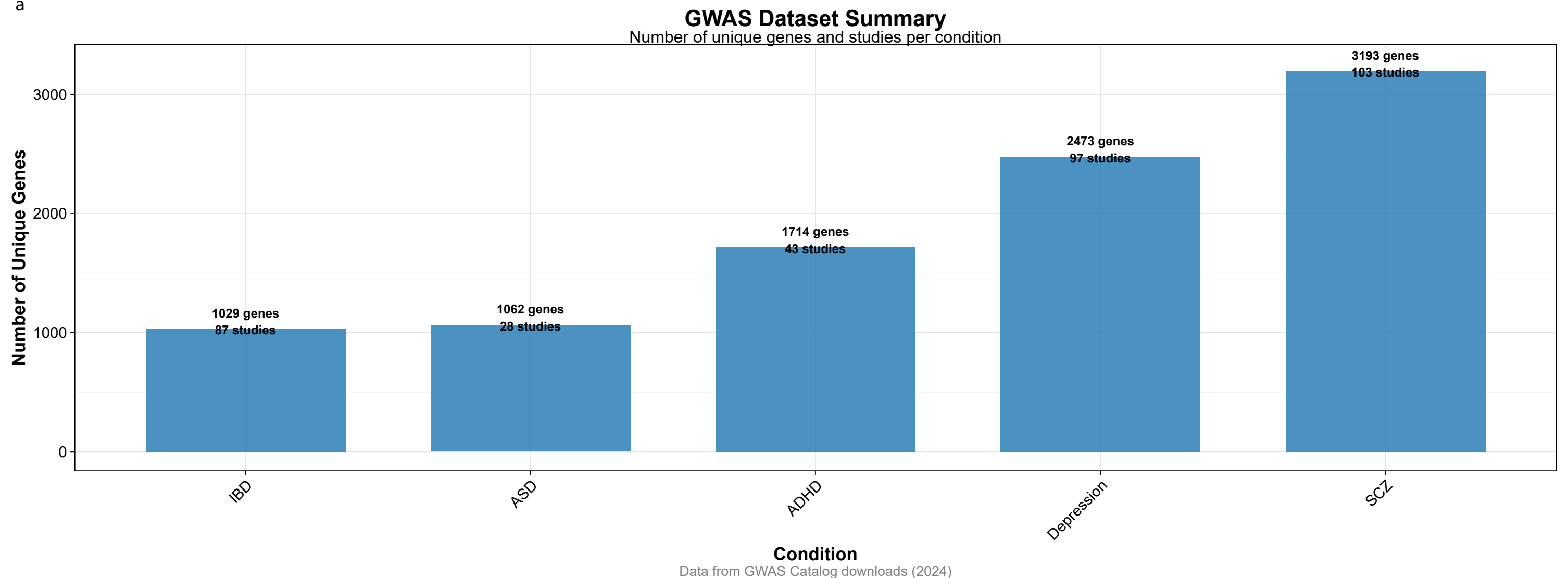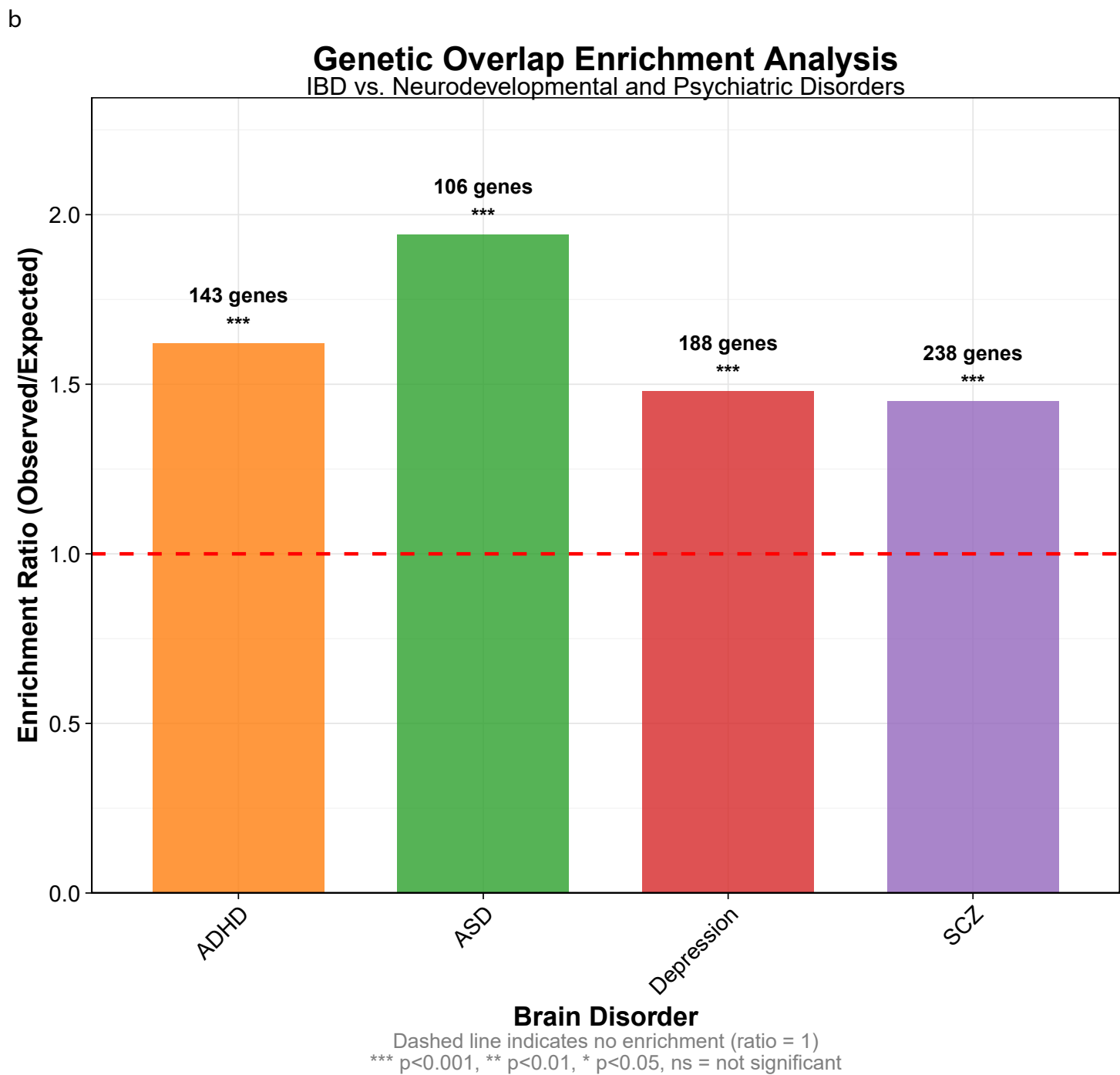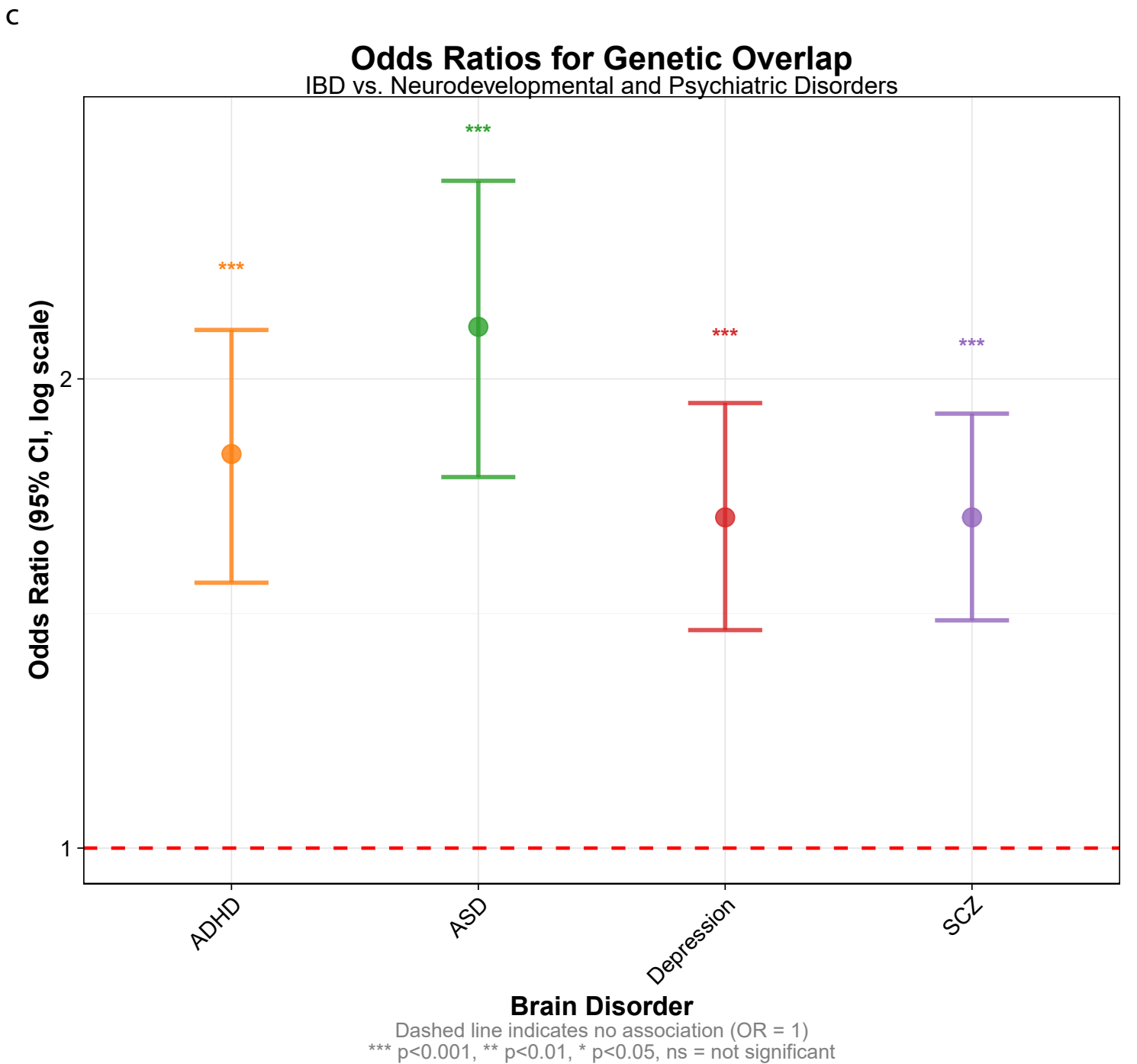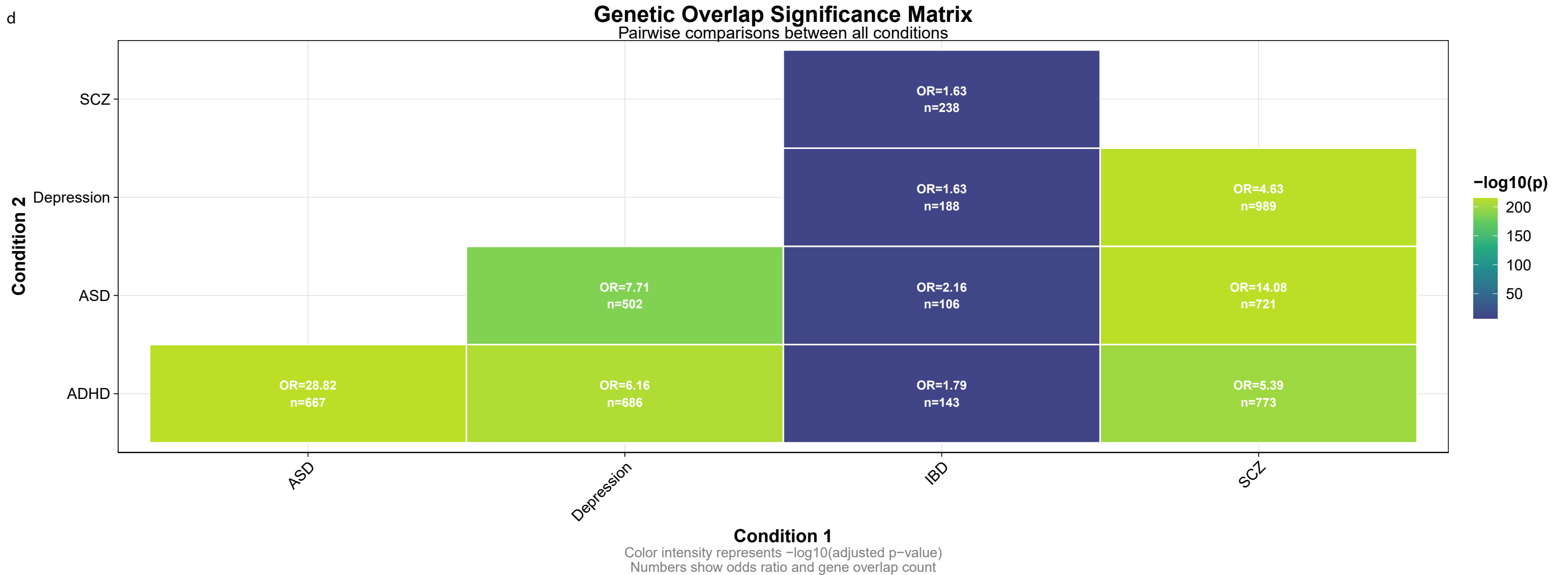

Supplement: Supplementary file 1 [file biology-14-01433-s001.zip › biology-3789580-supplementary/Supplemmentary figure S1.pdf]

a

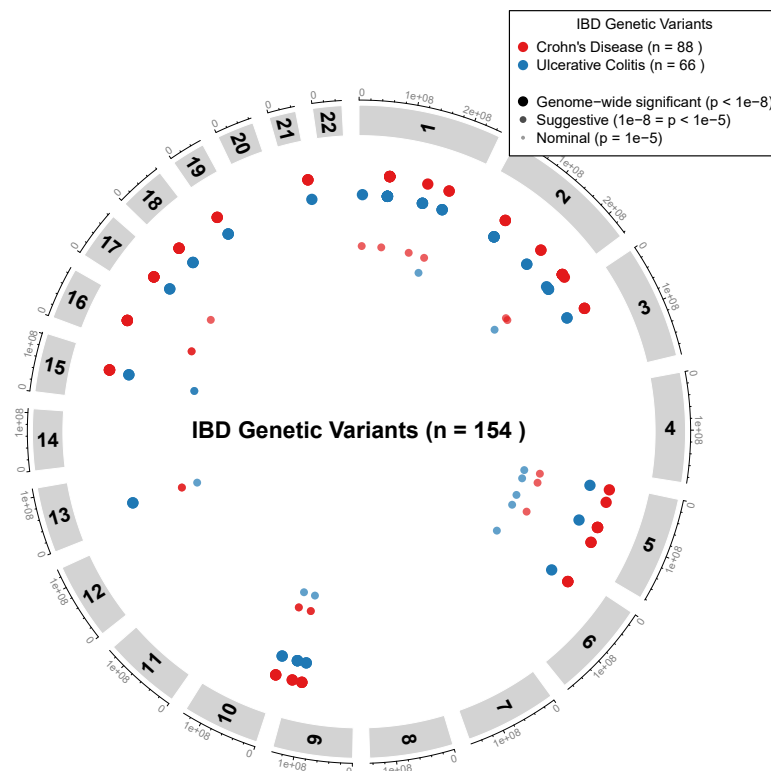

b

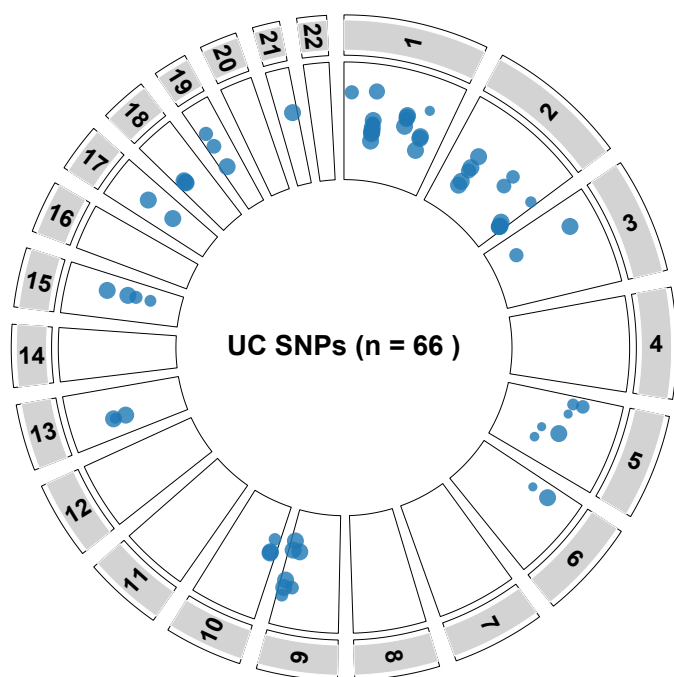

c

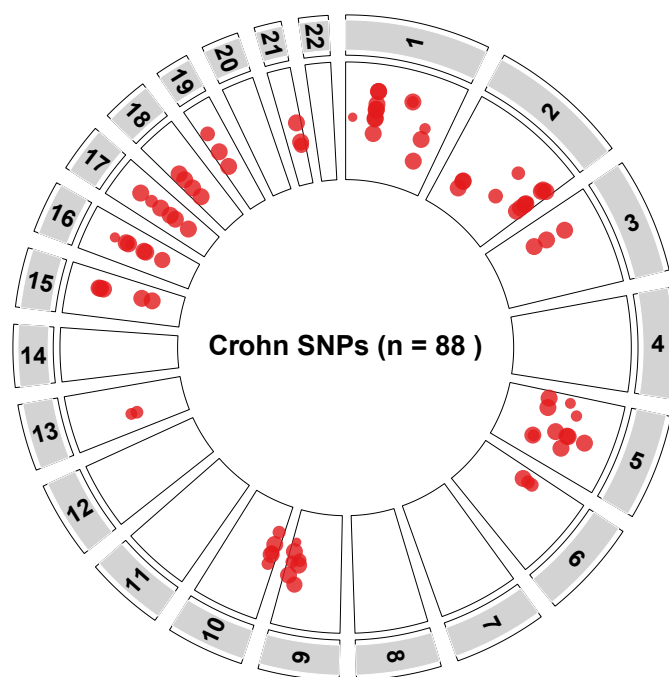

Supplement: Supplementary file 1 [file biology-14-01433-s001.zip › biology-3789580-supplementary/Supplemmentary figure S2.pdf]

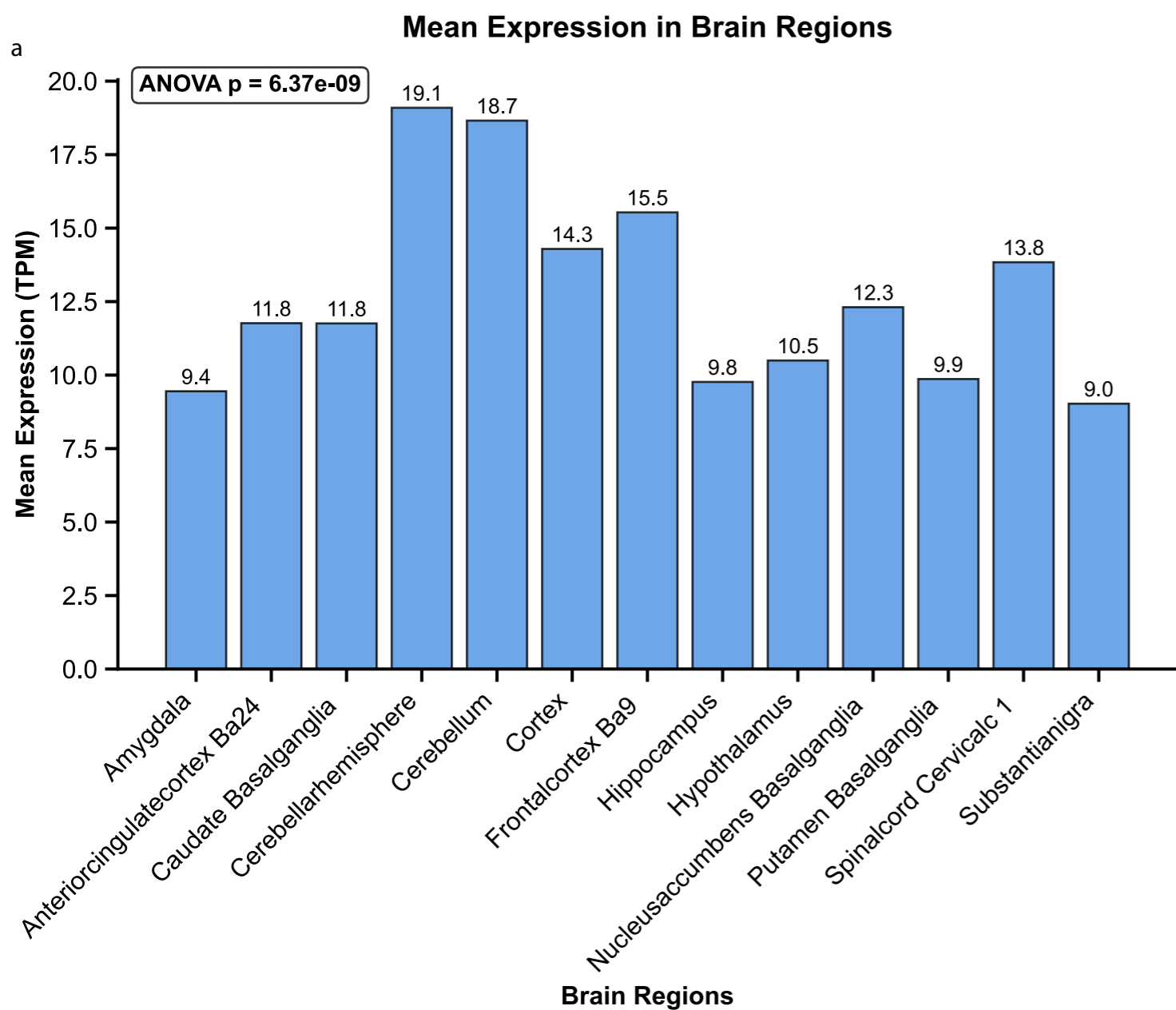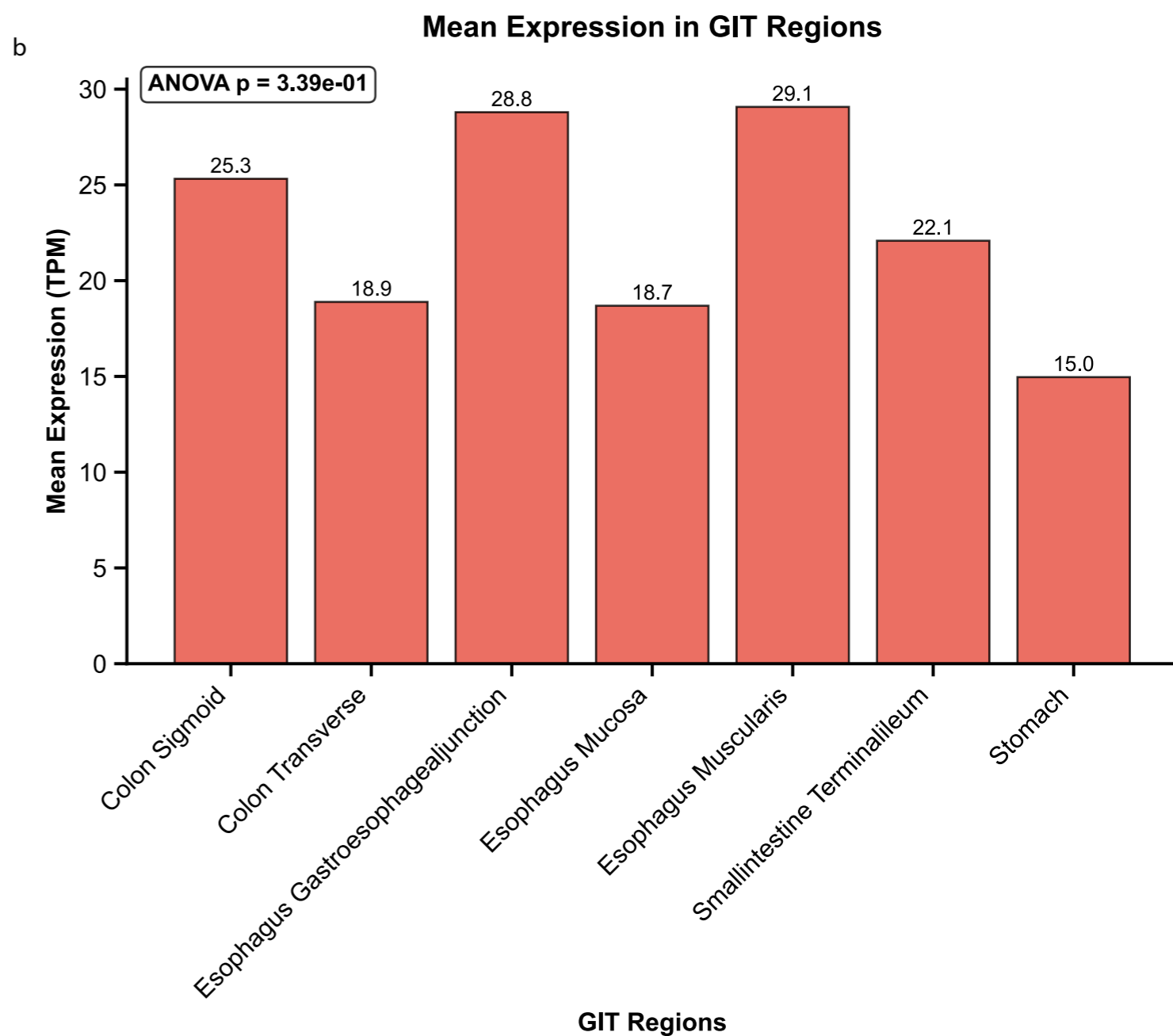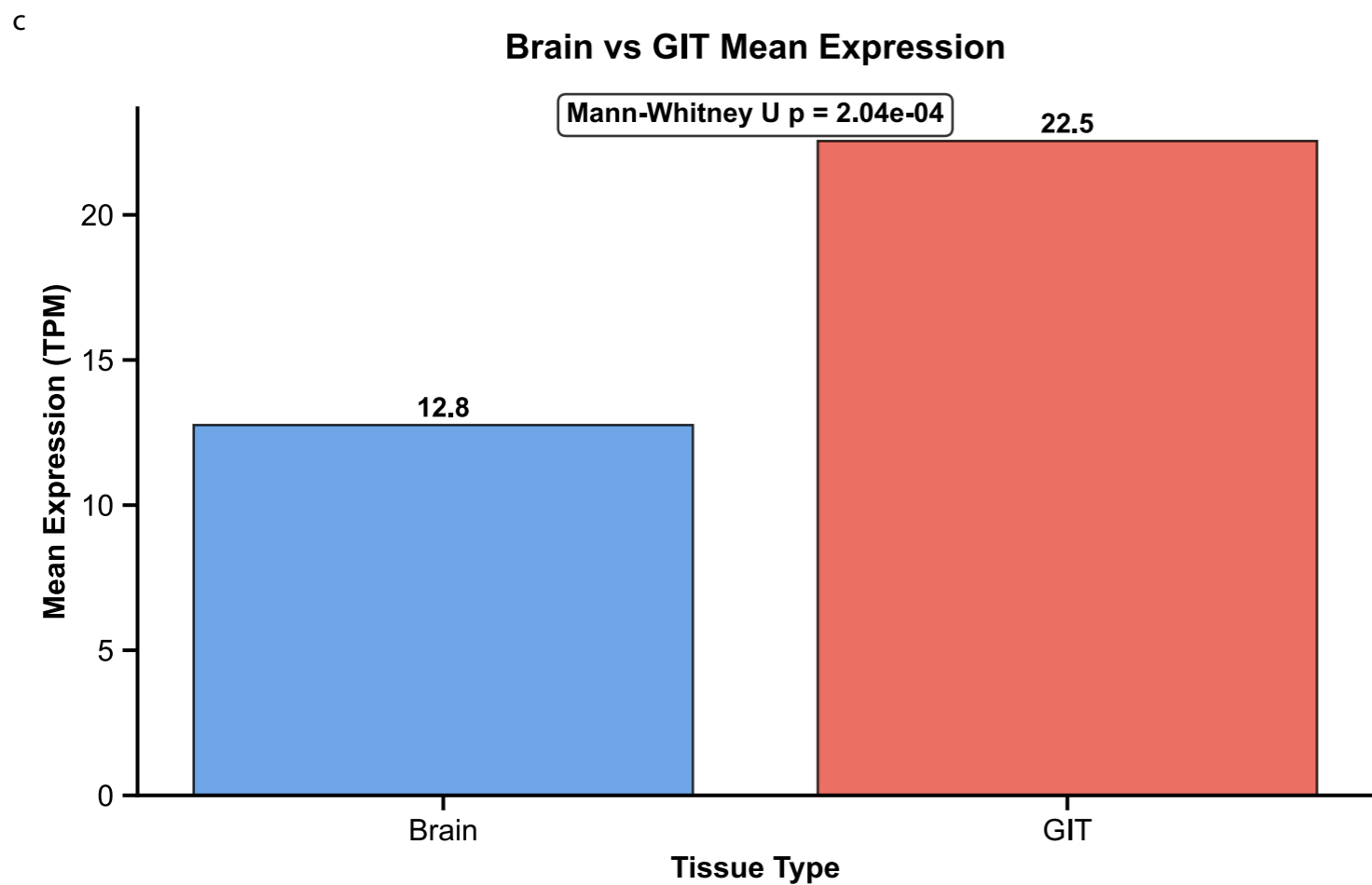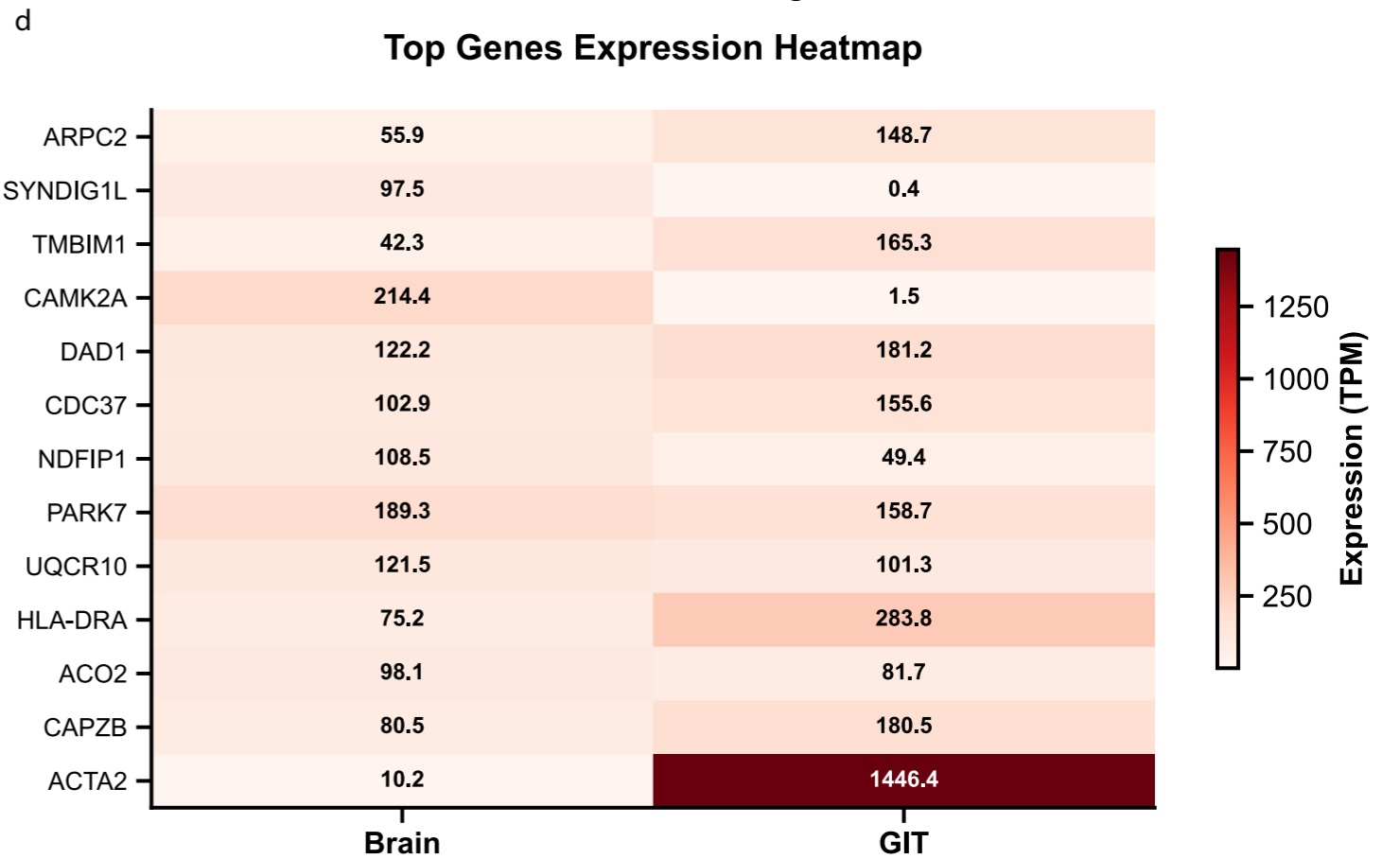

Supplement: Supplementary file 1 [file biology-14-01433-s001.zip › biology-3789580-supplementary/Supplemmentary figure S3.pdf]
